# Tuning structure and activity of propane dehydrogenation catalysts using tailored PtSn alloys

**DOI:** 10.1039/d6sc02757a

**Published:** 2026-08-10

**Authors:** Nestor Iwanojko, Enzo Brack, Domenico Gioffrè, Kazutaka Sakamoto, Christophe Copéret

**Affiliations:** a Department of Chemistry and Applied Biosciences (D-CHAB), ETH Zürich Zürich 8093 Switzerland ccoperet@inorg.chem.ethz.ch

## Abstract

The emergence of shale gas reserves as feedstock has necessitated the development of active and stable dehydrogenation catalysts to produce light olefins through alkane dehydrogenation. While PtSn catalysts are the industrial benchmark for propane dehydrogenation (PDH) processes, their underlying structure remains elusive. In this work, we apply surface organometallic chemistry (SOMC), combined with a thermolytic molecular precursor (TMP) to prepare a family of bimetallic PtSn catalysts supported on SiO_2_. We start with preparing isolated Sn/SiO_2_ sites *via* grafting and thermolysis of Sn(OSi(O^*t*^Bu)_3_)_4_, and characterize them *via* Fourier Transform Infrared Spectroscopy (FTIR), ^119^Sn solid state NMR and X-ray absorption spectroscopy (XAS). These well-defined Sn/SiO_2_ sites are subsequently used to prepare several bimetallic PtSn/SiO_2_ propane dehydrogenation catalysts with varying Pt : Sn ratios *via* grafting of a Pt precursor on Sn/SiO_2_, followed by temperature programmed reduction in hydrogen – yielding supported, alloyed Pt_*x*_Sn_*y*_ nanoparticles exhibiting ordered Pt_*x*_Sn_*y*_ phases as confirmed by powder X-ray diffraction (XRD). All prepared PtSn catalysts show remarkable PDH activity at a competitive weight hourly space velocity (WHSV) of 65 g_(C_3_H_8_)_/g_(Cat)_/h. The best catalyst, at a Pt : Sn ratio of 0.68, stabilizes at a productivity of 53 600 mol_(C_3_H_6_)_/mol_(Pt_Exposed)__/h after 30 h on stream, and we observe that it is primarily composed of the Pt_1_Sn_1_ alloy. Overall, our findings provide a strategy for the preparation of well-defined, supported Pt_*x*_Sn_*y*_ catalysts with tailored Pt : Sn ratios, while the formation of an alloyed Pt_1_Sn_1_ phase is identified to be key towards highly active and stable PtSn based PDH catalysts.

## Abstract 2

Development of a tailored synthesis methodology for PtSn propane dehydrogenation catalysts on silica shows that their performance is heavily depndent on the specific phase of the PtSn alloys formed.

## Graphical abstract

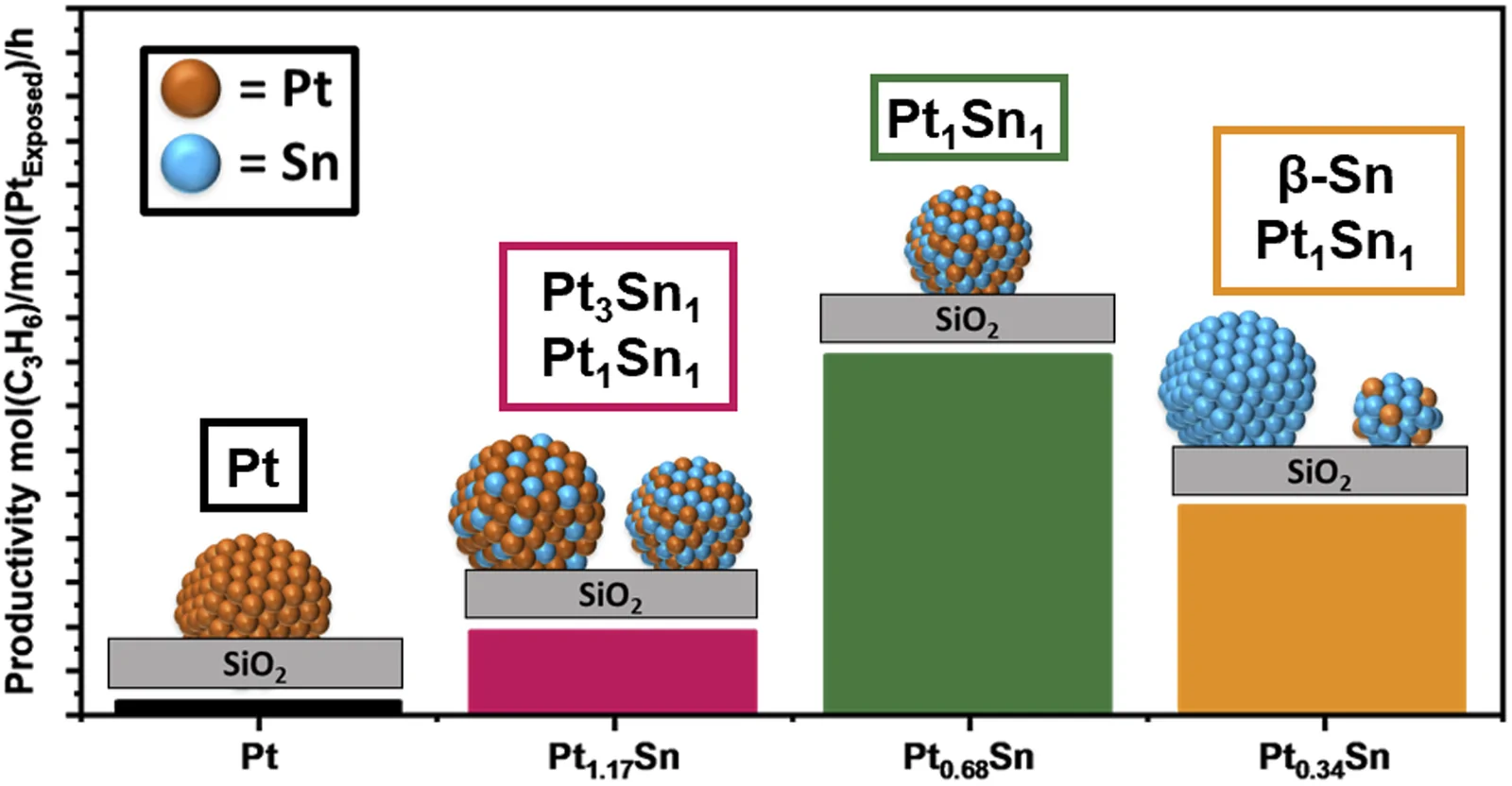

## Introduction

Access to large shale gas reserves has renewed the interest to produce light olefins *via* dehydrogenation as an alternative route to naphtha cracking.^1,2^ Due to thermodynamic constraints, on-purpose propane dehydrogenation (PDH) processes need to operate at high temperatures (>550 °C) and rely on catalysts, mostly based on Cr- or Pt.^3–5^ Catalyst deactivation *via* coke formation and/or sintering are particularly challenging aspects of these processes, requiring constant regeneration cycles and highly stable systems.^6,7^ One of the most established catalysts, applied in the UOP OLEFLEX process, utilizes an Al_2_O_3_-supported Pt based catalyst in combination with Sn promotion.^4^ Despite its widespread use, the promotional role of Sn in Pt-based PDH catalysts remains elusive, questioning the role of Sn interfacial sites, Sn oxide domains, and/or PtSn alloying in improving catalytic performance.^6^ It remains unclear whether the alloy formation causes improved performances or if Sn is only needed to merely dilute the active phase, consequently minimizing coke formation.^8^ Notably, the most common hypothesis links the presence of Sn to the formation of segregated Pt sites, thereby inhibiting coke formation and slowing down catalyst deactivation.^9^ A recently developed zeolite supported Pt single atom catalyst further supports this hypothesis, even though the role of Sn in such systems remains to be conclusively determined.^10–12^ However, in industrial applications, the formation of PtSn alloys has been reported as a key factor for catalyst stability.^13^ Mössbauer and XRD studies have identified the simultaneous presence of Pt_1_Sn_1_ and Pt_3_Sn_1_ phases as well as Sn(iv) and Sn(ii) species in PtSn catalysts prepared *via* incipient wetness impregnation (IWI).^14–18^ While Pt_3_Sn_1_ alloys have been claimed to yield the best activity,^19–22^ other reports describe the Pt_1_Sn_1_ phase as the best performing alloy.^23^ These reports identify specific alloy phases or Sn species present in active catalysts. However, a methodology able to access only one of the alloy phases, in the absence of residual Pt or Sn species, has remained a goal because such complexity has made it particularly challenging to decouple the role of tailored PtSn alloy phase(s) on catalyst performances. Conventional synthesis leads to a mixture of Sn species hindering the identification of stable catalytically active sites. Moreover, the low sensitivity of Sn Mössbauer and powder XRD of small nanoparticles makes it difficult to go beyond the identification of mixed phases.

Over the past decades, Surface Organometallic Chemistry (SOMC) has emerged as a powerful methodology for the preparation of tailored catalysts with controlled composition and interfaces.^24^ In a typical procedure, a thermolytic molecular precursor (TMP) is reacted with surface hydroxy groups of a partially dehydroxylated oxide support (*e.g.* SiO_2_, Al_2_O_3_, TiO_2_) in an organic solvent, thereby allowing for precise control over the distribution of metal sites and loading due to the absence of dissolution/precipitation processes that readily occur in aqueous media. Subsequent thermal treatment leads to the formation of isolated metal sites, nanoparticles (NPs), or any combination of these depending on the choice of precursor and/or reaction conditions.^24,25^ Notably, the SOMC methodology has been successfully employed for the preparation and study of bimetallic, SiO_2_-supported Pt-based PDH catalysts from isolated Ga(iii), Mn(ii) and Zn(ii) sites on SiO_2_ or Al_2_O_3_ to unveil the promotional role of dopants.^26–29^

The promotional role of Sn has so far not been investigated *via* SOMC. Alternative model systems were prepared *via* the direct and selective reaction of SnBu_4_ on the surface of SiO_2_-supported Pt NPs in the context of isobutane dehydrogenation.^9^ While alloy formation was confirmed *via* extended X-ray absorption fine structure (EXAFS) analysis, specific structure–activity relationships were not discussed.^30^ More recent studies have explored a co-grafting approach on *θ*-Al_2_O_3_ for the preparation of highly active PtSn nanoclusters albeit not addressing the roles of PtSn alloys in driving catalyst performance.^31^

Here, we develop a stepwise SOMC approach based on tetrakis(tris-*tert*-butoxysiloxide)Sn, Sn(OSi(O^*t*^Bu)_3_)_4_, a thermolytic molecular precursor. Grafting on silica followed by calcination generates SiO_2_-supported isolated Sn(iv) sites, Sn/SiO_2_. From Sn/SiO_2_, a series of bimetallic alloyed PtSn/SiO_2_ NPs, are prepared *via* subsequent grafting of Pt(η^3^-allyl)((*N-N*′-diisopropyl)acetamidinate) (Pt(η^3^-allyl)(DIA)) followed by a thermal treatment under a flow of H_2_. The SOMC approach allows for the preparation of well-defined catalysts with less complex and easier to study structures. Notably, these materials show no residual Sn sites or oxide domains and their PDH activity is governed by the Pt : Sn ratio, highlighting the formation of a Pt_1_Sn_1_ phase as a key factor towards active and stable PDH performance.

## Results & discussion

Isolated Sn sites supported on SiO_2_ (Sn/SiO_2_) are prepared *via* sequential grafting and calcination of a monomeric Sn(iv) molecular precursor, Sn(OSi(O^*t*^Bu)_3_)_4_ (Fig. 1a), which is prepared by reaction of Sn(NMe_2_)_4_ with HOSi(O^*t*^Bu)_3_ (ref. 32) or the reaction of SnCl_4_ with KOSi(O^*t*^Bu)_3_ (see SI for details). Grafting Sn(OSi(O^*t*^Bu)_3_)_4_ on partially dehydroxylated SiO_2–700_ (1.0 equiv. ≡Si–OH/nm^2^) through protonolysis of the Sn–O bond (details in SI) yields supported Sn sites, Sn(OSi(O^*t*^Bu)_3_)_*n*_@SiO_2_, as confirmed by elemental analysis (EA) (2.45 w% Sn and 2.78 w% C). This corresponds to *ca.* 0.55 Sn/nm^2^ and 1 equiv. of –OSi(O^*t*^Bu)_3_ ligand remaining on the surface per grafted Sn precursor which implies decomposition of –OSi(O^*t*^Bu)_3_ moieties on the surface as analogously reported for Ga(OSi(O^*t*^Bu)_3_)_3_(THF).^33^ The preparation of Sn/SiO_2_ is followed *via* IR spectroscopy (Fig. 1b), showing the consumption of Si–OH groups at 3745 cm^−1^ and the concomitant appearance of C–H stretches associated with the –OSi(O^*t*^Bu)_3_ ligand at 3000–2750 cm^−1^, consistent with grafting of Sn(OSi(O^*t*^Bu)_3_)_4_*via* protonolysis. Notably, the presence of a broad feature centered at 3350 cm^−1^ in the FTIR spectrum of Sn(OSi(O^*t*^Bu)_3_)_*n*_@SiO_2_, indicates the interaction of residual silanols with the immobilized Sn precursor, *e.g.* the –OSi(O^*t*^Bu)_3_ ligand. Subsequent thermal treatment under a flow of synthetic air (SA) at 600 °C leads to the formation of isolated Sn sites, Sn/SiO_2_ free of organic residues, as well as the restoration of isolated ≡Si–OH groups (3745 cm^−1^) as seen from the FTIR spectrum of Sn/SiO_2_ (Fig. 1b). Notably, a sharp peak at 3662 cm^−1^ appears associated with the presence of isolated surface ≡Sn–OH species appears after calcination.^34^ In agreement, high-resolution high-angle annular dark-field (HAADF) scanning transmission electron microscopy (STEM) of Sn/SiO_2_ (SI Fig. S20) reveals the high dispersion Sn sites with no sign of aggregation. Moreover, EA confirms a retention of the Sn content upon thermal treatment (2.71 w% Sn, 0.55 Sn/nm^2^). The preparation of Sn/SiO_2_ is further monitored by ^119^Sn-ssNMR spectroscopy. The ^119^Sn-ssNMR spectrum of the Sn(OSi(O^*t*^Bu)_3_)_4_ precursor shows a peak at *δ*_iso_ = −455 ppm as expected for a tetrahedral Sn(iv) complex.^35,36^ Upon grafting, a signal centered at −667 ppm – up-field by ∼200 ppm with respect to the Sn precursor (Fig. 1c) – is detected, indicating that Sn(iv) adopts an octahedral environment upon grafting, likely through interaction with adjacent siloxane bridges and/or residual OH groups.^37,38^ The ^119^Sn ssNMR spectrum of Sn/SiO_2_ shows a broad signal with a maximum at −483 ppm and shoulder at −646 ppm. Upon deconvolution of the two signals, we propose a *ca.* 3 : 1 mixture of tetrahedral and octahedral Sn(iv) sites for Sn/SiO_2_ considering that both are expected to have similar relaxation times, but one cannot exclude the possibility of a continuum of various Sn sites (SI Fig. S4).

**Fig. 1:**
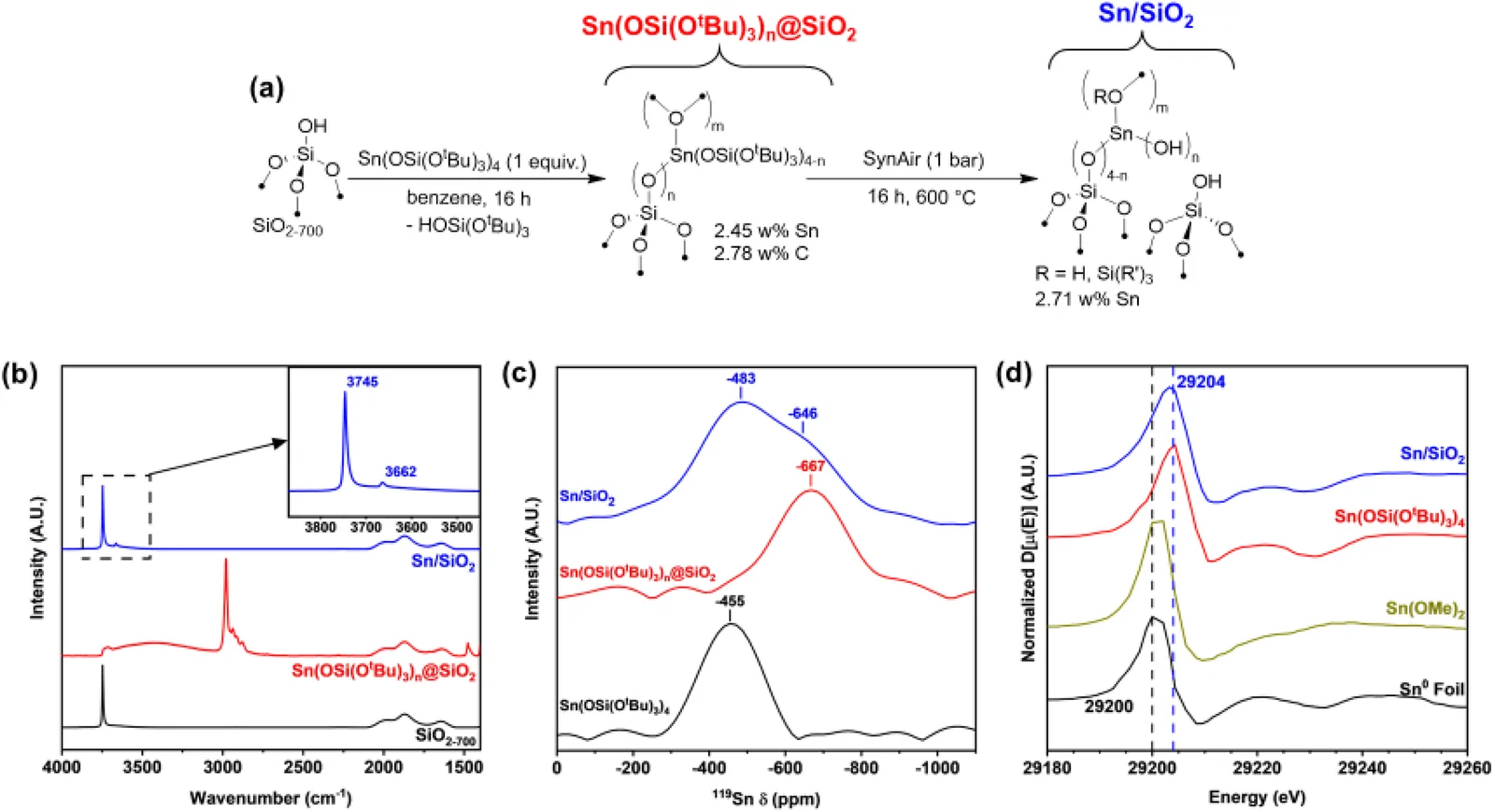
(a) Preparation of Sn sites on SiO_2_*via* a SOMC/TMP approach. (b) FTIR spectra and (c) static ^119^Sn-ssNMR of Sn(OSi(O^*t*^Bu)_3_)_4_ (black), grafted Sn(OSi(O^*t*^Bu)_3_)_4_@SiO_2_ (red), and Sn/SiO_2_ (blue). (d) First derivative XANES spectra at the Sn-K edge of Sn Foil (black), Sn(OMe)_2_ (gold), Sn(OSi(O^*t*^Bu)_3_)_4_ (red), and Sn/SiO_2_ (blue).

To further interrogate the state of Sn in Sn/SiO_2_, the X-ray Absorption Near Edge Structure (XANES) spectrum of the material at the Sn K-edge is acquired and compared to those of Sn foil, Sn(OMe)_2_, and Sn(OSi(O^*t*^Bu)_3_)_4_ (Fig. 1d). Notably, the edge energy of Sn/SiO_2_ (29 204 eV) is identical to the one of Sn(OSi(O^*t*^Bu)_3_)_4_ (Sn(iv), 29 204 eV), and higher than both Sn foil (Sn(0), 29 200 eV) and Sn(OMe)_2_ (Sn(ii), 29 202 eV), indicating that the Sn sites on Sn/SiO_2_ correspond to Sn(iv), consistent with observations from ^119^Sn-ssNMR. Next, Pt(η^3^-allyl)((*N-N*′-diisopropyl) acetamidinate) (Pt(η^3^-allyl)(DIA)) is reacted with the surface OH groups of Sn/SiO_2_, producing a pale-yellow material. Subsequent thermal treatment at 600 °C under a flow of hydrogen leads to the formation of a black material, coined Pt_*x*_Sn where *x* represents the Pt : Sn atomic ratio determined from EA (Fig. 2a). To tailor the PtSn alloy composition, three bimetallic Pt–Sn materials with varying Pt : Sn ratios are prepared by varying the Pt loadings aiming for a series of 1 : 1, 1 : 2, and 1 : 3 Pt : Sn ratios. The metal content of the final materials is determined by EA to Pt_1.17_Sn (4.05 w% Pt, 2.06 w% Sn), Pt_0.68_Sn (2.31 w% Pt, 2.09 w% Sn), and Pt_0.34_Sn (1.17 w% Pt, 2.06 w% Sn) (Table 1) and the amount of exposed Pt is determined with CO chemisorption. A monometallic Pt reference catalyst, Pt/SiO_2_ NPs (*ca.* 2 nm), is also prepared using the same approach, using SiO_2–700_ as a support in place of Sn/SiO_2_.^39,40^ Notably, monitoring the preparation of these materials by IR spectroscopy reveals the grafting of the Pt precursor on the surface OH groups and subsequent removal of all organic ligands upon H_2_ treatment at 600 °C (Fig. 2b).

**Fig. 2:**
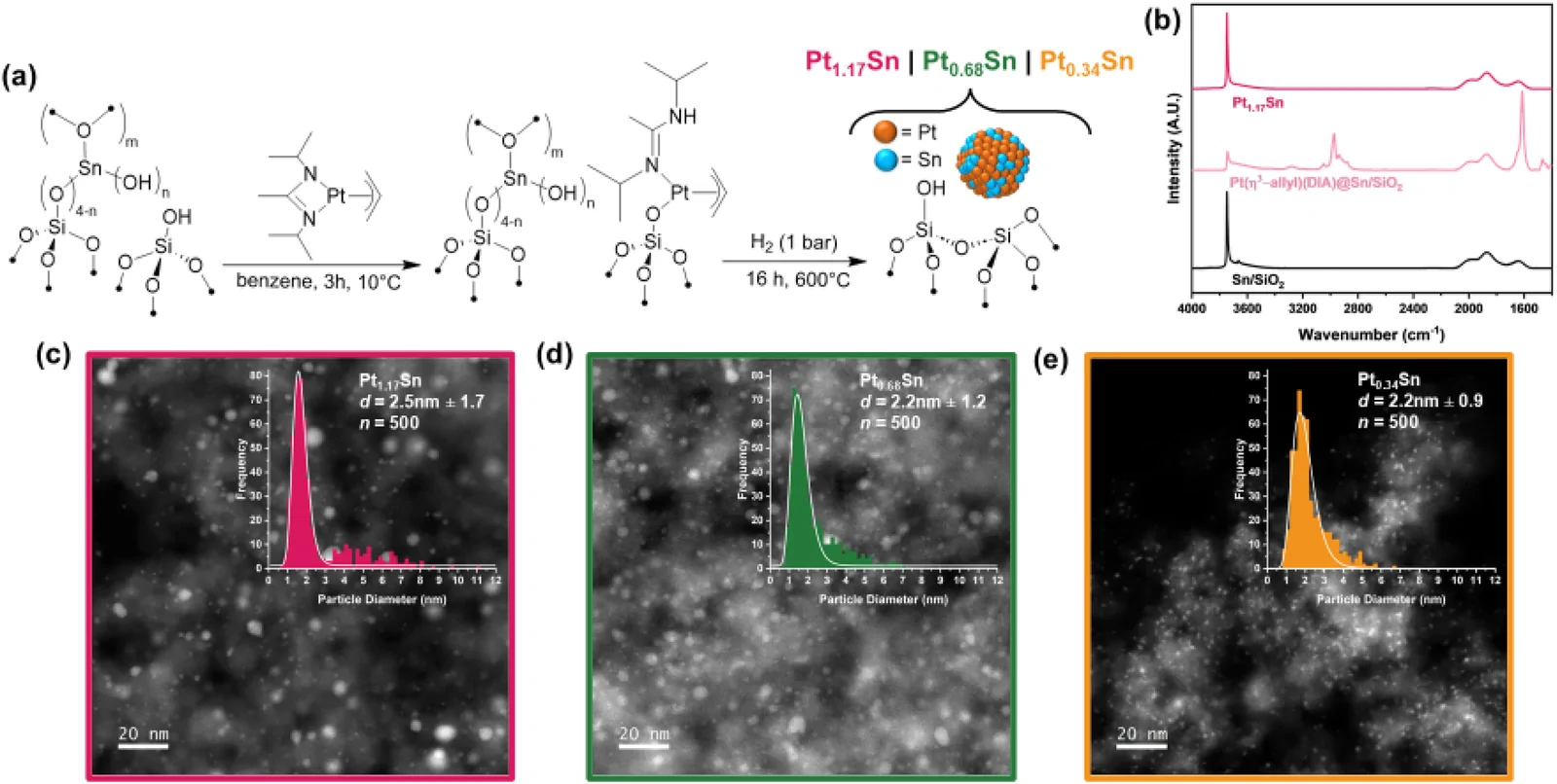
(a) Preparation of bimetallic SiO_2_ supported PtSn NPs from Sn/SiO_2_*via* SOMC. (b) Sequential FTIR spectra of Sn/SiO_2_ (black), grafted Pt(η^3^-allyl)(DIA)@Sn/SiO_2_ (pink), and Pt_1.17_Sn (red). Representative HAADF STEM images and corresponding particle size distributions of (c) Pt_1.17_Sn, (d) Pt_0.68_Sn, and (e) Pt_0.34_Sn.

**Table 1:** Elemental analysis, metal dispersion, Pt : Sn ratio, particle size (obtained from STEM), and µmol Pt accessible per g obtained by CO chemisorption of the PtSn catalysts and Pt/SiO_2_ reference

Material	Elemental analysis (w%)		Atom per nm^2^		Pt : Sn ratio	Size (nm)	CO chemisorption (µmol Pt_Exposed_ g^−1^)
	Pt	Sn	Pt	Sn			
Pt_1.17_Sn	4.05	2.06	0.56	0.47	1.17	2.5 ± 1.7	18.05
Pt_0.68_Sn	2.31	2.09	0.32	0.47	0.68	2.2 ± 1.2	2.72
Pt_0.34_Sn	1.17	2.06	0.16	0.47	0.34	2.2 ± 0.9	0.57
Pt/SiO_2_	3.96	—	0.55	—	—	2.0 ± 1.0^35^	23.78

HAADF STEM imaging (Fig. 2c–e) of the various PtSn catalysts at constant Sn loadings shows the formation of small particles with a size distribution of 2.5 nm ± 1.7 (Pt_1.17_Sn), 2.2 nm ± 1.2 (Pt_0.68_Sn), and 2.2 nm ± 0.9 (Pt_0.34_Sn) respectively. The size distribution appears to become narrower across samples as less Pt is added. Pt_1.17_Sn also appears to have a bimodal size distribution and hints at the presence of two sets of Pt_*x*_Sn_*y*_ particles (*vide infra*). STEM energy dispersive X-ray spectroscopy (EDXS) mapping of the as-prepared catalysts shows clearly overlapping Pt M and Sn L maps, suggesting alloying of Pt and Sn in all three materials (SI Fig. S22–S24).

XANES spectra at the Pt L_3_-and Sn K-edges indicate that for all bimetallic materials, both Pt and Sn are in the metallic state. The Sn K-edge (Fig. 3a) energy of all bimetallic catalysts matches the edge energy of Sn foil at 29 200 eV, suggesting no remaining Sn(ii) or Sn(iv) sites. Similarly, the Pt L_3_-edge energy, remains the same across all bimetallic samples with 11 565 eV (Fig. 3b), close to Pt(0) foil (11 564 eV), consistent with alloying of PtSn.^41,42^ A similar effect was previously observed for other PtM bimetallic M-promoted PDH catalysts (M = Mn, Ga, and Zn).^27,28,33^

**Fig. 3:**
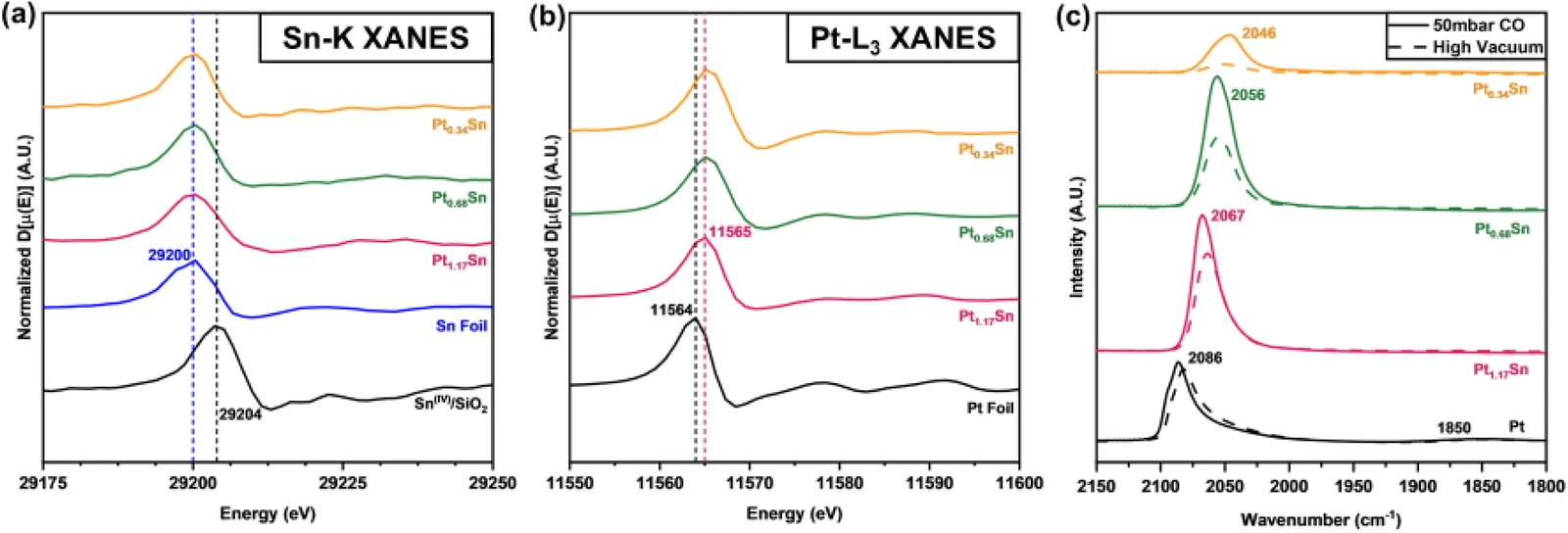
(a) First derivative XANES spectra at the Sn-K edge of Sn/SiO_2_ (black), Sn foil (blue), Pt_1.17_Sn (red), Pt_0.68_Sn (green), Pt_0.34_Sn (yellow). (b) First derivative XANES spectra at the Pt-L_3_ edge of Pt foil (black), Pt_1.17_Sn (red), Pt_0.68_Sn (green), Pt_0.34_Sn (yellow). (c) CO adsorption FTIR spectra of Pt/SiO_2_ (black), Pt_1.17_Sn (red), Pt_0.68_Sn (green), and Pt_0.34_Sn (yellow), a spectrum of the pristine material is subtracted as a blank.

The coordination environment of Pt is further examined *via* Pt L_3_-edge EXAFS analyses (SI Fig. S31). In all three cases, the best fit is achieved when including both Pt–Pt and Pt–Sn paths, further supporting the formation of PtSn alloys across the prepared catalysts (Table 2). The coordination numbers for Pt_1.17_Sn, Pt_0.68_Sn, and Pt_0.34_Sn are 5.7, 2.2, and 2.3 for Pt–Pt and 3.2, 4.5, and 4.7 for Pt–Sn respectively.

**Table 2:** Pt-L_3_ EXAFS fitting parameters with the scattering path length (*r*), mean square relative displacement (Debye–Waller-factor) (*σ*^2^), and change in internal energy alignment (Δ*E*_0_)

Material	Path	Coordination numbers	*r* (Å)	*σ* ^2^ (Å)	Δ*E*_0_ (eV)
Pt_1.17_Sn	Pt–Pt	5.7 ± 0.6	2.78 ± 0.01	0.0075 ± 0.0016	2.34 ± 0.68
	Pt–Sn	3.2 ± 0.3	2.69 ± 0.01		
Pt_0.68_Sn	Pt–Pt	2.2 ± 0.5	2.77 ± 0.03	0.0051 ± 0.0013	3.28 ± 0.98
	Pt–Sn	4.5 ± 0.6	2.69 ± 0.01		
Pt_0.34_Sn	Pt–Pt	2.3 ± 0.9	2.78 ± 0.04	0.0052 ± 0.0022	2.61 ± 1.58
	Pt–Sn	4.7 ± 0.9	2.68 ± 0.02		

When more Pt is added to the system the coordination number of the Pt–Pt path increases and the Pt–Sn path decreases suggesting that Pt_1.17_Sn is composed of a comparatively Pt rich alloy with respect to the other catalysts. Notably, the coordination numbers of the Pt–Pt and Pt–Sn path for Pt_0.68_Sn and Pt_0.34_Sn are nearly the same, suggesting that these materials are composed of the same types of alloys, while Pt_1.17_Sn has a different alloy composition. In addition, the nearest neighbor Pt : Sn ratios derived from the coordination numbers of Pt–Pt and Pt–Sn are 1.8, 0.5, and 0.5 for Pt_1.17_Sn, Pt_0.68_Sn, and Pt_0.34_Sn respectively, suggesting that Pt_1.17_Sn contains more Pt rich nanoparticles. Finally, the total coordination number (Pt–Pt + Pt–Sn) drops from 8.9 for Pt_1.17_Sn to 7.0 for Pt_0.34_Sn, consistent with the decrease in particle size as observed *via* HAADF STEM imaging.

To further understand the effect of PtSn alloying on the electronic structure of the nanoparticles, FTIR spectra of all catalysts are acquired after exposure to CO (50 mbar) as well as after subsequent evacuation under high vacuum (10^−4^ mbar) (Fig. 3c).

A consistent redshift of 19 cm^−1^, 30 cm^−1^, and 40 cm^−1^, with respect to the monometallic Pt NPs, is found for Pt_1.17_Sn, Pt_0.68_Sn, and Pt_0.34_Sn respectively, in the linear CO peak as more Sn is introduced to the system. In addition, the bridging CO feature is not detectable in the bimetallic catalysts. This further supports the formation of alloys with different compositions. Evacuation of the CO adsorption FTIR cell slightly decreases the peak intensity for Pt_1.17_Sn and Pt_0.68_Sn, thereby indicating reversible adsorption of some of the chemisorbed CO. In sharp contrast, most of the CO is reversibly bound for Pt_0.34_Sn, indicating weak adsorption of CO for the lowest Pt : Sn ratio.

While XAS and CO adsorption FTIR measurements hint at the presence of various alloys in these materials, the structure of the bimetallic particles as a function of Pt : Sn ratio remains unknown. Thus, we turn to XRD measurements to identify the bulk structure of specific Pt_*x*_Sn_*y*_ phases (see SI for further discussion). Indeed the simultaneous presence of both Pt_3_Sn_1_ ^43,44^ and Pt_1_Sn_1_ ^44,45^ is detected in Pt_1.17_Sn. As seen with EXAFS, Pt_1.17_Sn does contain more Pt rich alloys. Notably, only Pt_1_Sn_1_ is detected for Pt_0.68_Sn (Fig. 4a). Given the Pt : Sn ratios in the materials *vs.* the structure identified *via* XRD, we propose that the excess Sn remains in metallic β-Sn domains considering that it is not incorporated into well-defined alloys. Crystalline particles of these alloys are also observed *via* high-resolution HAADF STEM imaging, while their respective *d*-spacing matches that of the structures observed from XRD. For Pt_1.17_Sn, crystalline nanoparticles exhibiting Pt_3_Sn_1_ and Pt_1_Sn_1_ phases are observed (Fig. 4b), while for Pt_0.68_Sn only the Pt_1_Sn_1_ phase is formed (Fig. 4c). For lower Pt content as in Pt_0.34_Sn, we additionally observe the presence of large β-Sn domains as judged by the narrow peaks in the XRD pattern, matching the reported β-Sn structure,^44,46^ further supporting the claim that excess Sn remains metallic. However, broad peaks are also seen at 2*θ* = 15° and 27°, consistent with the presence of small Pt_1_Sn_1_ nanoparticles in agreement with observations from HAADF STEM imaging (Fig. 4d).

**Fig. 4:**
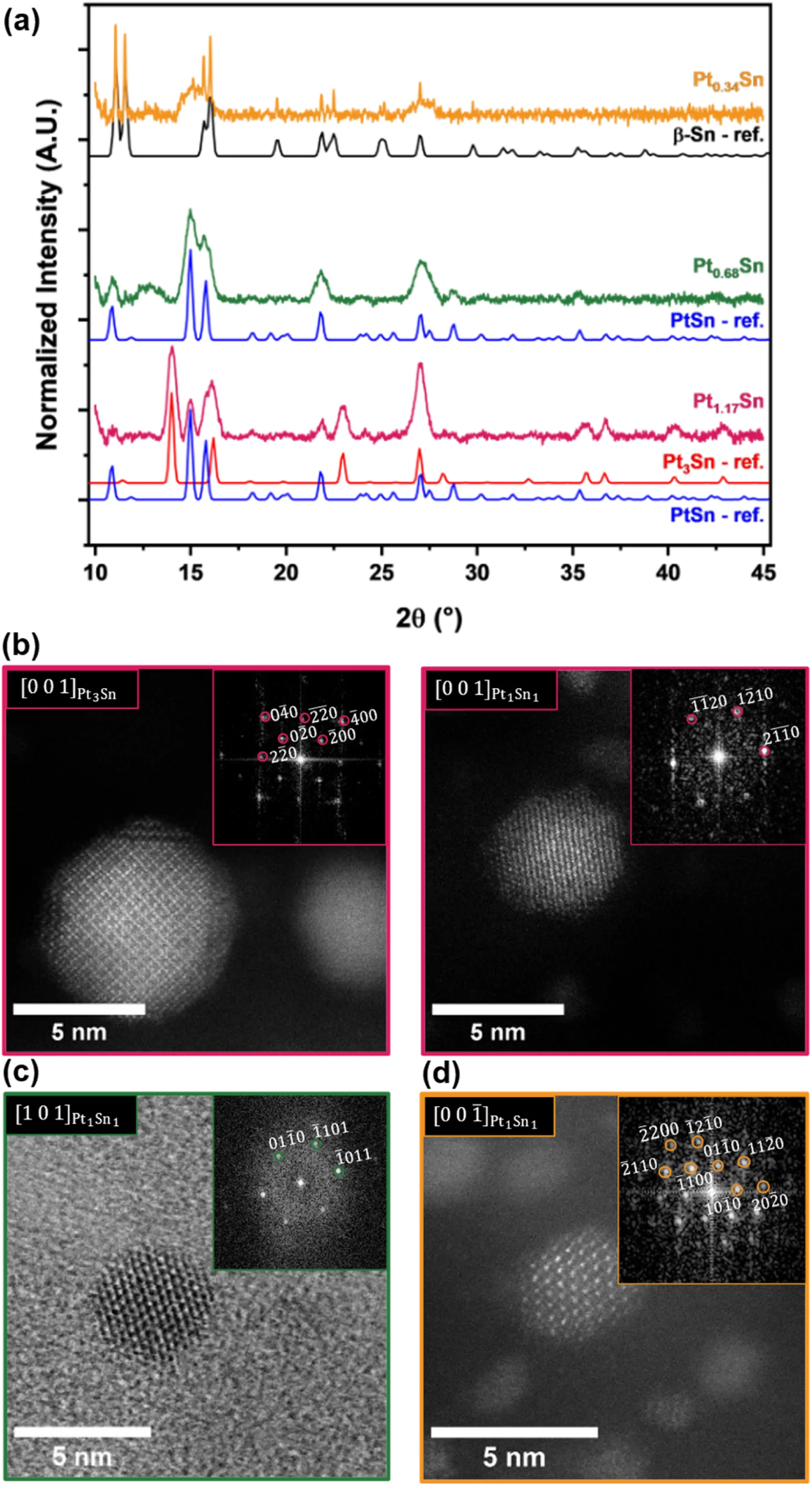
(a) XRD patterns (*λ* = 0.56325 Å) of Pt_1_,_17_Sn, Pt_0.68_Sn, and Pt_0.34_Sn along with their matching reference patterns below each measurement. Reference patterns are given for Pt_3_Sn_1_ (red, ICSD-105795 (ICSD release 2026.1)), Pt_1_Sn_1_ (blue, ICSD-42594 (ICSD release 2026.1)), and β-Sn (black, ICSD-252800 (ICSD release 2026.1)). High-resolution HAADF STEM images along with the FFT of crystalline nanoparticles of Pt_1.17_Sn (b) Pt_0.68_Sn (c) and Pt_0.34_Sn (d) exhibiting both Pt_3_Sn_1_ and Pt_1_Sn_1_ phases (b) or only a Pt_1_Sn_1_ phase (c) and (d). The FFT inlay is matched to the referenced alloy phase and reported as [U V W]_Pt_*x*_Sn_*y*__.

All bimetallic materials are next tested for PDH at 550 °C (1 bar(g) of pressure with a 50 mL min^−1^ flow composed of 20% C_3_H_8_ in argon at a WHSV = 62 g_(C_3_H_8_)_/g_(Cat)_/h) and compared to Pt/SiO_2_ (Fig. 5). Notably, at high Pt loading, Pt_1.17_Sn suffers from relatively fast deactivation (50% loss in conversion after 30 hours on stream). In contrast, Pt_0.68_Sn and Pt_0.34_Sn (Fig. 5a and Table 3) are significantly more stable with only a *ca.* 5% loss of conversion over 30 h. Additionally, a 2 hour activation period is observed for Pt_0.68_Sn and Pt_0.34_Sn, indicating the occurrence of dynamic changes under reaction conditions to form the active phases. Normalizing the productivity by the amount of accessible Pt as determined by CO chemisorption (mol_(C_3_H_6_)_/mol_(Pt_Exposed_)_/h) reveals that all bimetallic catalysts exhibit significantly higher activity per Pt with respect to monometallic Pt (Fig. 5b) with maximum productivities of 18 200, 81 000, 46 700, 2 100 mol_(C_3_H_6_)_/mol_(Pt_Exposed_)_/h for Pt_1.17_Sn, Pt_0.68_Sn, Pt_0.34_Sn, and Pt respectively. Notably, Pt_0.68_Sn and Pt_0.34_Sn, both associated with the Pt_1_Sn_1_ phase, display higher productivity than Pt_1.17_Sn which exhibits both a Pt_3_Sn phase and Pt_1_Sn_1_ phase. After 30 h on stream, Pt_0.68_Sn and Pt_0.34_Sn also exhibit the best stability retaining 66% and 84% of their maximum productivity respectively, while Pt_1.17_Sn drops to 45% of its maximum activity (See SI for fitting of deactivation rates & parameters).^47^ Overall, we observe a volcano trend in the Pt : Sn ratio with Pt_0.68_Sn exhibiting the best activity.

**Fig. 5:**
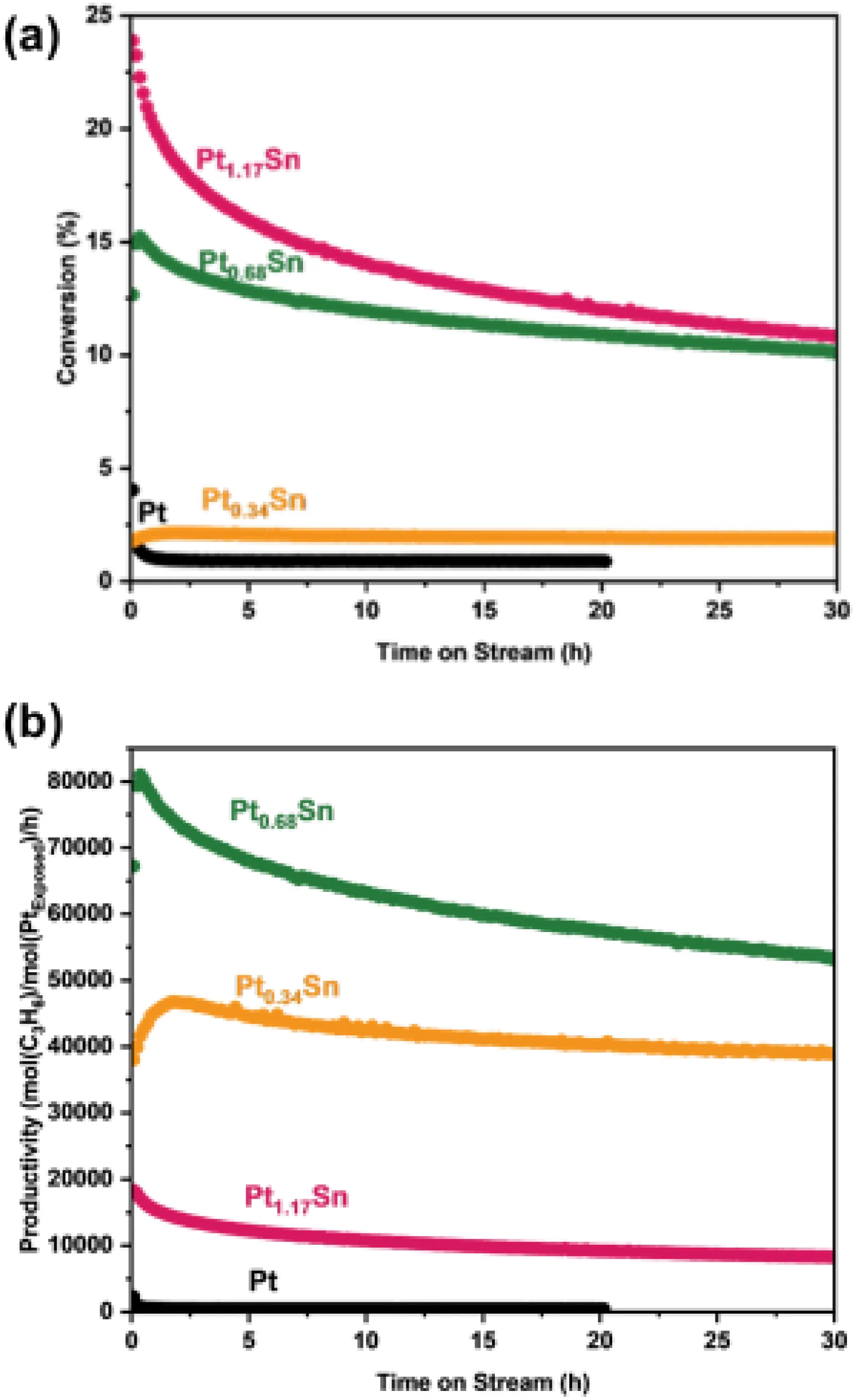
(a) Conversion (%) and (b) productivity (mol_(C_3_H_6_)_ mol_(Pt_Exposed_)_ h^−1^) over time for Pt/SiO_2_ (black), Pt_1.17_Sn (red), Pt_0.68_Sn (green), and Pt_0.34_Sn (yellow).

**Table 3:** Catalytic parameters of the PDH tests for supported PtSn systems

Material	Time (h)	Pt loading (w%)	Conversion (%)	C_3_H_6_ Selectivity (%)	Productivity (mol_(C_3_H_6_)_/mol_(Pt_Exposed_)_/h)
Pt	0.2	3.96	1.76	82.17	867
	2		0.90	64.24	347
Pt_1.17_Sn	0.2	4.05	23.24	99.02	17 745
	2		18.38	98.90	14 019
	30		10.78	98.06	8211
Pt_0.68_Sn	0.2	2.31	14.91	98.67	79 387
	2		13.81	98.52	73 423
	30		10.09	97.63	53 635
Pt_0.34_Sn	0.2	1.17	1.82	88.27	39 884
	2		2.11	89.25	46 643
	30		1.87	84.42	39 229

Considering the difference in catalytic performances across the series, STEM EDXS measurements are carried out post-catalytic test (after 30 h on stream), showing a retention of the PtSn speciation with no evidence of dealloying (SI Fig. S26–S28). Notably, only Pt_1.17_Sn shows signs of sintering while the size distribution of Pt_0.68_Sn and Pt_0.34_Sn remains unchanged post-catalysis (SI Fig. S25). Significant carbon coverage in the EDXS mappings of the post-catalysts suggests the main cause for catalyst deactivation is coke coverage. Correlating the Pt_*x*_Sn_*y*_ phases with the catalytic PDH performance, shows that the Pt_3_Sn_1_ alloy phase seems more prompt to deactivate as judged from the comparatively fast deactivation of Pt_1.17_Sn. In addition the broader particle size distribution only present for Pt_1.17_Sn suggests that the larger bimetallic particles could be responsible for this faster deactivation. Meanwhile the presence of a Pt_1_Sn_1_ alloy phase is key to high catalytic activity and catalyst stability as seen in the best performing Pt_0.68_Sn catalyst.

## Conclusion

Isolated Sn-sites are prepared from a thermolytic molecular Sn(OSi(O^*t*^Bu)_3_)_4_ precursor *via* a combined SOMC/TMP approach and characterized *via* FTIR, XANES and ^119^Sn solid-state NMR. Subsequently, tailored bimetallic PtSn PDH catalysts with varying Pt : Sn ratios are prepared using a Pt(η^3^-allyl)(DIA) molecular precursor *via* a SOMC approach. Their composition is tuned by adjusting the Pt : Sn ratio to give Pt_1.17_Sn (4.04 w% Pt), Pt_0.68_Sn (2.06 w% Pt) and Pt_0.34_Sn (1.17 w% Pt). The formation of PtSn alloys is confirmed *via* XAS at the Sn K- and the Pt L_3_-edge, STEM EDXS mapping and CO-IR. Notably, XRD and high-resolution HAADF STEM imaging support the concomitant formation of alloyed Pt_3_Sn_1_ and Pt_1_Sn_1_ phases for Pt_1.17_Sn, while only a Pt_1_Sn_1_ phase is detected for Pt_0.68_Sn. With lower Pt content, additionally to a Pt_1_Sn_1_ phase, β-Sn domains are formed as observed for Pt_0.34_Sn.

Correlating the PDH performance with the observed PtSn phases highlights the importance of a Pt_1_Sn_1_ phase in driving catalyst activity and stability in PDH, while the presence of a Pt_3_Sn_1_ phase induces faster deactivation, pointing towards Pt site isolation as origin of the promotional role of Sn in PtSn PDH catalysts. Overall, our findings point out the need to control the nature of the PtSn alloy to design robust and high performing PDH catalysts.

## Author contributions

NI: investigation, formal analysis, writing – original draft, writing – review and editing, conceptualization; EB: investigation, formal analysis, writing – review and editing; KS, DG, and CC: conceptualization, formal analysis, supervision, writing – review and editing.

## Conflicts of interest

The authors declare no conflicts of interest.

## Supplementary Material

SC-017-D6SC02757A-s001

## Data Availability

The data supporting this article have been included as part of the supplementary information (SI). Supplementary information is available. See DOI: https://doi.org/10.1039/d6sc02757a.
