# Supplementary material for: Tuning structure and activity of propane dehydrogenation catalysts using tailored PtSn alloys
Source: Chem Sci. 2026 Aug 10;17(37):18027–34. doi: 10.1039/d6sc02757a (PMC13456048; doi:10.1039/d6sc02757a)
Supplement: Supplementary file 1 [file SC-017-D6SC02757A-s001.pdf]

Supplementary Information (SI) for Chemical Science.
This journal is © The Royal Society of Chemistry 2026

Supporting Information: Tuning Structure and Activity of Propane Dehydrogenation Catalysts using Tailored PtSn Alloys

Nestor Iwanojko, Enzo Brack, Domenico Gioffrè, Kazutaka Sakamoto, Christophe Copéret*

Department of Chemistry and Applied Biosciences (D-CHAB), ETH Zürich, Zürich, 8093, Switzerland

*E-mail: ccoperet@inorg.chem.ethz.ch

Contents

1 General 0

2 Characterization 1

3 Synthetic Methods 5

4 Catalyst Evaluation 7

4.1 Propane Dehydrogenation Catalytic Tests: 7

4.1 Analysis of Catalyst Deactivation 8

5 Supporting Figures and Tables 10

5.1 NMR Spectroscopy: 10

5.2 Chemisorption Analysis 14

5.3 Electron Microscopy: 16

5.4 X-Ray Absorption Spectroscopy: 22

5.5 Attempt at Rietveld refinement of pXRD data 23

6 References 24

1 General

Basic procedures:

All experiments were performed under inert argon atmosphere using standard Schlenk, glovebox, and high vacuum techniques (10^{-5} mbar) unless mentioned otherwise. Solvents such as n-pentane and n-heptane were dried by passage through double M-Braun SPS alumina solvent purification columns. Tetrahydrofuran (THF) and benzene were dried and freshly distilled under argon from purple Na^0/benzophenone. Deuterated benzene was vacuum distilled from Na^0/benzophenone. All solvents were stored over activated 4Å molecular sieves prior to use. Molecular sieves and celite were activated overnight at 320°C under high vacuum (10^{-5} mbar). The molecular complexes $Sn(OMe)_2$[1], Pt(AMD)η^3-allyl[2], were prepared according to literature procedures. The synthesis of $Sn(NMe_2)_4$ and $Sn(OSi(O^tBu)_3)_4$ are given below. All other reagents were purchased from Sigma-Aldrich or Strem Chemicals and used as received. Solution ^1H, ^{13}C, and ^{119}Sn NMR spectra were recorded in Teflon J. Young valve-sealed NMR tubes on a Bruker 200 or 300 MHz spectrometer and the chemical shifts are referenced in ppm related to the solvent. Solid state ^{119}Sn NMR spectra were recorded on a Bruker 400 MHz DNP spectrometer and the chemical shifts were cross referenced to SnO_2 powder at -604 ppm.

2 Characterization

Elemental analysis:

All elemental analyses were performed at the Mikroanalytisches Labor Pascher (Pulvermühle 1, D-53424 Remagen, Germany).

NMR-Quantification of Washing Solution:

A ^{1}H NMR spectrum of the washing solution along with a ferrocene standard (typically around 30 mg) is measured. The moles of precursor that are left over and failed to graft are calculated based on the integrations of the known ferrocene and leftover precursor integrals providing an estimate of the metal weight loading. For NMR-scale grafting, 5mg of ferrocene was used (Figure S15).

Fourier Transform Infrared Spectroscopy (FTIR):

All IR spectra were recorded in transmission mode on a Bruker ALPHA spectrometer and a Nicolet 6700 FT-IR spectrophotometer with 1 cm^{-1} spectral resolution, in the range of 400 – 4000 cm^{-1} and an average of 32 scans.

CO Chemisorption:

Chemisorption experiments were carried out on a BELSORP-max apparatus (BelJapan). Approximately 190 mg of reduced catalyst was loaded in an airtight cell inside a glovebox which was then mounted on the apparatus. The sample was pre-treated at 300 °C for 1 h at 10−5 mbar. The chemisorption measurements were performed at 25 °C, the pressures at equilibrium were recorded when the pressure variation was below 0.6% for 1200 seconds. The molar uptake was calculated from measured volumetric uptake assuming ideal gas law at standard temperature and pressure. Metal dispersion analysis plots are given in Figure S16 - Figure S18.

CO-IR probe:

For the IR studies upon CO adsorption (CO-IR probe), the IR spectra were recorded from freshly prepared, self-supporting pellets (5-14 mg) of the studied materials. Self-holding pellets on an aluminum ring were placed inside an IR shuttle (glass vacuum cell) equipped with CaF_2 windows. The cell was first evacuated under high vacuum (10^{-5} mbar) and a blank spectrum of the material, prior to CO addition, was measured. After exposure to CO gas (10 mbar for ^{12}CO) another spectrum was recorded. A third spectrum was recorded after exposure to 50 mbar ^{12}CO. For some materials a spectrum at 100 mbar ^{12}CO was also measured. The final spectrum was collected after evacuation to 10^{-5} mbar for 10 minutes to investigate the disappearance of the CO bands from the reversibly bound CO molecules. For discussion, the background subtracted IR spectra are shown.

Electron Microscopy:

Scanning transmission electron microscopy (STEM) measurements were performed on an aberration-corrected JEOL JEM-ARM 300F microscope operated at 300 kV within the facilities of ScopeM at ETH Zurich. The samples were grinded with a plastic spatula inside an Ar Glove Box and subsequently dry casted onto a 400 mesh Cu grid coated with ultra-thin carbon on lacey carbon from TedPella and transferred to the microscope under an inert atmosphere, using a vacuum transfer holder from Gatan. The signals of the STEM energy dispersive x-ray spectroscopy (EDXS) maps were processed using an averaging filter.

Particle size measurements were performed using the ImageJ software[3] using darkfield images. Crystalline particles were analyzed using ImajeJ to set the scale of the image and process the FFT. Next, to determine the miller indicies, a simulated diffraction pattern was made using CrystalMaker[4] and a CIF file obtained from the Inorganic Crystal Structure Database (ICSD)[5]. The correct [U V W] orientation was set and the diffraction peaks from the simulated pattern were matched to those seen in the FFT of the crystalline particle.

X-Ray Absorption Spectroscopy:

X-ray absorption spectroscopy (XAS) experiments were measured at BM31 of the Swiss-Norwegian Beamlines (SNBL) located at the European Synchrotron Radiation Facility (ESRF) in Grenoble, France. Sn K-edge spectra were collected in transmission mode using a double crystal Si (111) monochromator. In each case, the ionization chambers were filled with a gas mixture optimized for the edge energy and the required path length. For each edge, a secondary reference for energy calibration was used (Sn-Foil). Typical beam dimensions used were 0.4 mm (H) x 4 mm (W); the beam size was controlled using slits. The sample was packed into quartz capillary. The powdered sample was secured using a quartz wool plug and sealed with wax and epoxy resin. For acquisition of EXAFS spectra, beam energies of 29 keV for the Sn K edge, were collected. 8 scans for Sn K edge to obtain a sufficient quality suitable for structural analysis. For acquisition of EXAFS spectra, beam energies of 13.879 keV for the Pt L_3 edge, were collected. 8 scans for Pt L_3 edge to obtain a sufficient quality suitable for structural analysis. XAS analysis was performed using the Demeter software package[6]. XANES spectra were analyzed using Athena and EXAFS fitting was performed using Artemis.

Synchrotron X-Ray Powder Diffraction Measurements:

Synchrotron X-Ray powder diffraction measurements (SXRD) were measured at BM01[7] of the Swiss-Norwegian beamlines (SNBL) located at the European synchrotron radiation facility (ESRF) in Grenoble, France. Without exposure to air, the sample was finely ground and packed into 0.1mm thick quartz capillary. The powdered sample was secured using a quartz wool plug and sealed with wax and epoxy resin. Measurements were in the range 2θ = (0.1,60) with λ = 0.56325 Å. Figure S1 shows the raw data along with the baseline correction. The processing is outlined below:

1) Raw data was imported into OriginPro2024 graphic and data analysis software[8].
2) All diffraction patterns were then normalized to the highest intensity peak.
3) Over the range of 2θ = (0.1,50), an asymmetric least squares (ALS) fitting was applied to construct a baseline with an Asymmetric factor of 0.001, threshold of 0.005, smoothing factor of 6 and 10 iterations.
4) The baseline was then subtracted from the raw data.
5) The range was cut to 2θ = (10,45) and the data was normalized again to the highest intensity peak.

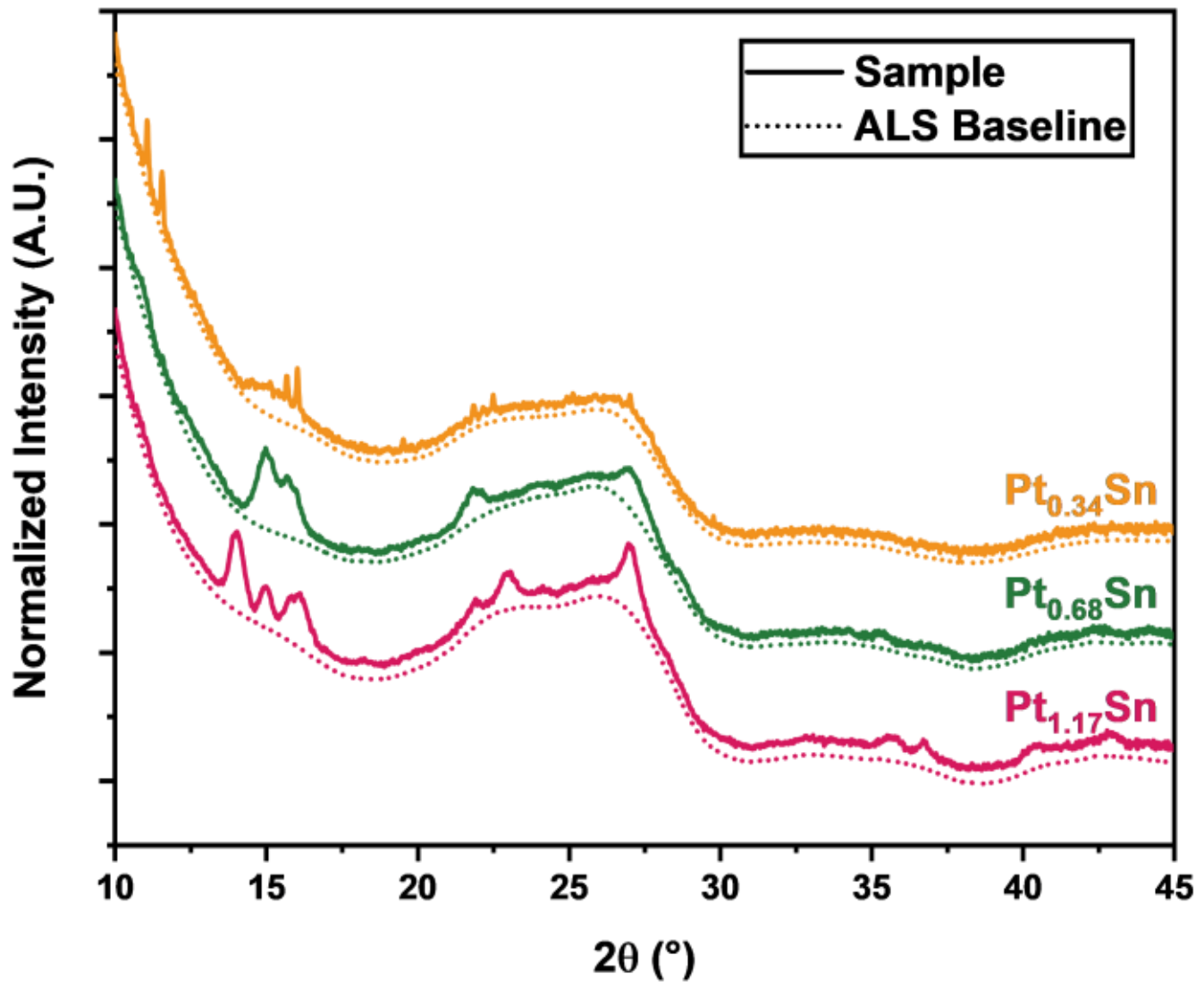

Figure S1. Raw SXRD patterns obtained from $Pt_{1.17}Sn$ (red), $Pt_{0.68}Sn$ (green), and $Pt_{0.34}Sn$ (yellow). Data is given as solid line, and baseline is given as dotted line.

Powder X-Ray Diffraction Measurements:

Powder X-Ray diffraction (PXRD) measurements were acquired on a high-precision two circle goniometer Stoe Stadi P, equipped with a curved Germanium (111) monochromator, an anti-scatter collimator and a Mythen2 R 3K detector. The same processing method was applied to the data as for the SXRD measurements. Data were acquired using a Cu source for 2θ = (20, 80.015) with a step length of 0.015 and 14.4 seconds per step. Figure S2S shows the raw data along with the baseline correction. Figure S3S shows the baseline subtracted PXRD patterns. The powder patterns produced by the Stoe Stadi P hinted at the presence of small, alloyed, and crystalline PtSn NPs, however, the pattern is overshadowed by large β-Sn domains. Therefore, we turned to SXRD to pick better detect the smaller crystalline domains.

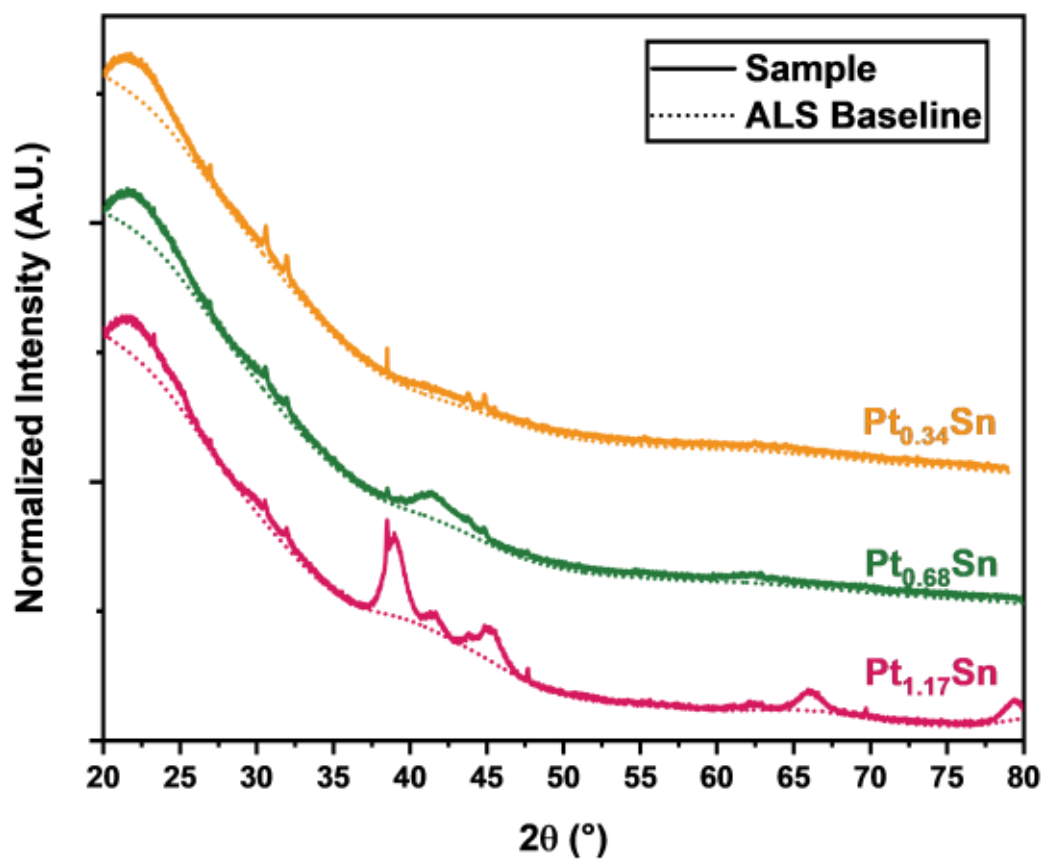

Figure S2. Raw PXRD patterns obtained from $Pt_{1.17}Sn$ (red), $Pt_{0.68}Sn$ (green), and $Pt_{0.34}Sn$ (yellow). Data is given as solid line, and baseline is given as dotted line.

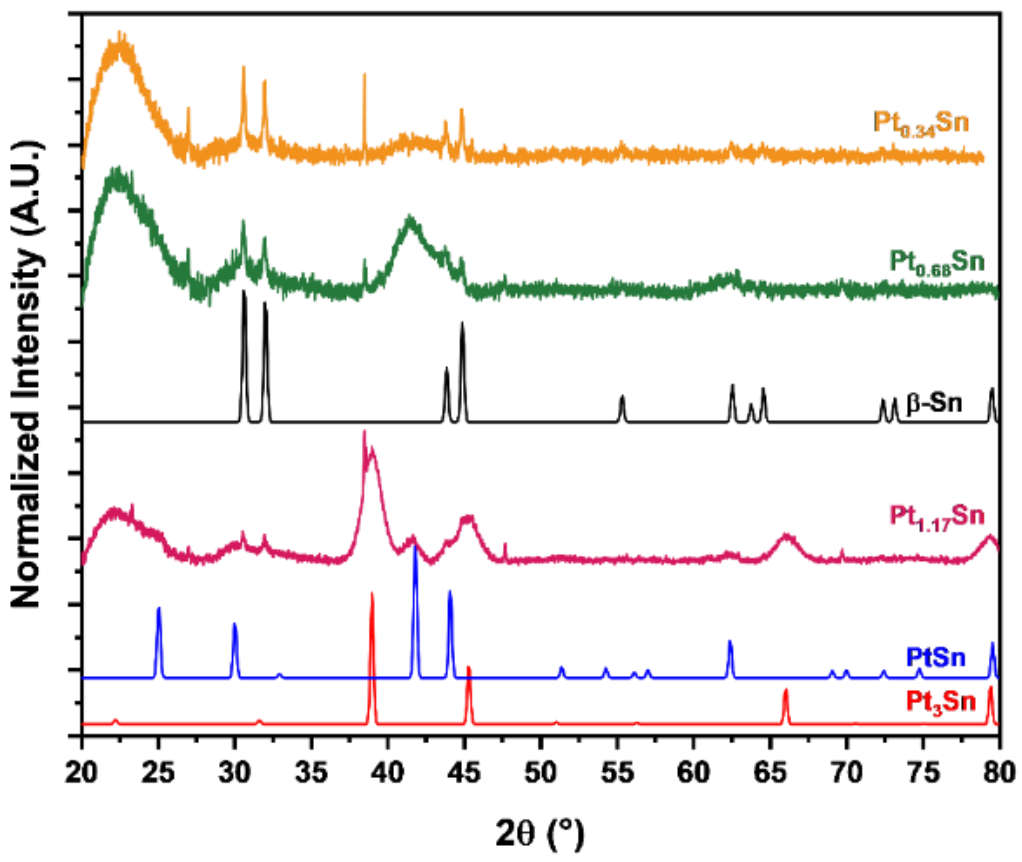

Figure S3. Baseline corrected PXRD patterns obtained from $Pt_{1.17}Sn$ (red), $Pt_{0.68}Sn$ (green), and $Pt_{0.34}Sn$ (yellow) along with references. Peaks at 2θ = 27° and 38° could not be assigned.

ssNMR Measurements:

Solid state ^{119}Sn NMR spectra were recorded on a Bruker AVANCE NEO 400 spectrometer (9.4 T) at low temperature (100 K), equipped with a 3.2-mm Bruker DNP MAS NMR probe. Thin-wall 3.2-mm zirconia rotors were employed. All static spectra were recorded using ultra-wideline NMR methodology[9], which employs advanced, frequency-swept pulse schemes (wideband, uniform rate, smooth truncation[10]) for increased excitation bandwidths[11], and multiple-echo acquisition (Carr–Purcell–Meiboom–Gill[12,13]) for sensitivity enhancement. Recycle delays of 0.5 s were used throughout. The final ^{119}Sn NMR spectra were obtained by coadding the individual echoes of the WCPMG-echo train in the time domain before Fourier transformation followed by magnitude calculation. The chemical shifts were cross referenced to SnO_2 powder at -604 ppm. For Sn/SiO_2 the spectrum was deconvoluted using Gaussian fits shown in Figure S4S, resulting in the 3:1 ratio of tetrahedral to octahedral sites.

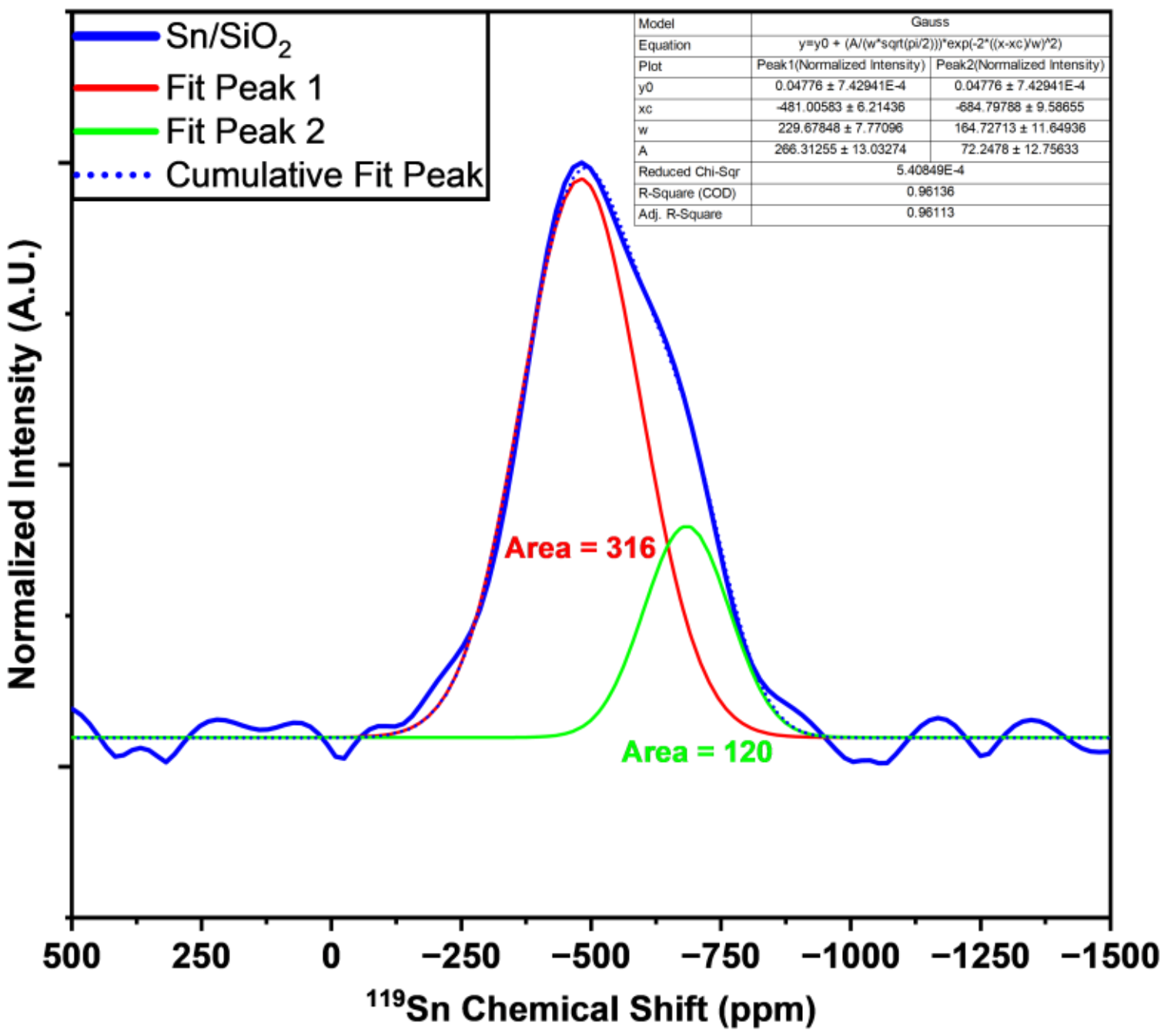

Figure S4. Static ^{119}Sn-ssNMR spectrum of Sn/SiO_2 along with the fitting for the two proposed types of Sn sites. Peak 1 (red) is assigned to tetrahedral sites and Peak 2 (green) is assigned to octahedral sites.

3 Synthetic Methods

Preparation of silica support:

Silica support, partially dehydroxylated at 700 °C (SiO2−700), was prepared by heating Degauss Aerosil (204 m^2/g) to 500 °C (5 °C/min), calcining in air for 8 hours, evacuating to high vacuum (10^{-5} mbar), maintaining a temperature of 500 °C for 8 hours, heating to 700 °C (1 °C/min), and maintaining 700 °C for 20 hours. ^{1}H NMR titration of the SiO_{2-700} using $Mg(CH_2Ph)_2$, yielded 0.32 – 0.36 mmol OH g^{-1} corresponding to 0.9 accessible OH groups per nm^2.

Synthesis of $Sn(NMe_2)_4$: This is a modification of the literature procedure of Jones et.al.[14]. To a 3-neck roundbottom (250 mL) fitted with a reflux condenser, n-pentane (20 mL) and ether (10 mL) were added. Next, $SnCl_4$ (1.2 mL, 10.10 mmol) was added followed by a slurry of $LiNMe_2$ (2.24 g, 42.42 mmol) in ether (45 mL) which was delivered *via* cannula transfer. The mixture was set to reflux for 4 hours and then left to cool overnight while stirring. The supernatant was filtered out and the salts were washed with ether (3 × 10 mL). After this step, the solution was filtered a second time paying close attention to avoid picking up the precipitated LiCl salt. A slightly yellow solution was obtained and the solvent was removed *en vacuo*. The product was then vacuum distilled at 58 °C to give 1.2 mL (47%) of a clear slightly oily liquid. Stored in the freezer under Argon. Some unreacted $SnCl_4$ could be identified in the ^{119}Sn NMR. The compound was then used without further purification.

^{1}H NMR (300 MHz, C_6D_6): *δ* 2.80 (24H). ^{13}C NMR (75 MHz, C_6D_6): *δ* 43.24. ^{119}Sn NMR (112 MHz, C_6D_6): -120.1 ($Sn(Nme_2)_4$), -146.2 ($SnCl_4$).

Synthesis of $Sn(OSi(O^tBu)_3)_4$: A n-pentane (25 mL) solution of tris(tert-butoxy)silanol (4.24 g, 16.04 mmol) was cannula transferred to a n-pentane (15 mL) solution of $Sn(NMe_2)_4$ (1.0 mL, 3.96 mmol) while stirring at 0 °C. The clear solution turned a cloudy white and the mixture was left to stir overnight at room temperature. The solvent was then removed *en vacuo* and the solid was left to sublime overnight on high vacuum (10^{-5} mbar) and room temperature. After one day of sublimation, the ^{1}H NMR showed some leftover $HOSi(O^tBu)_3$, therefore the solid was left to sublime on high vacuum for another night. This gave a crude yield of 3.85 g (82.9%). Next, the resulting white powder was recrystallized in n-pentane at -40 °C giving long white crystals. ^{1}H NMR of the crystals shows 98% purity vs. leftover $HOSi(O^tBu)_3$.

^{1}H NMR (300 MHz, C_6D_6): δ 1.54 (108H), 1.39 (HOTBOS 2%). ^{13}C NMR (75 MHz, C_6D_6): δ 32.3, 72.9. ^{119}Sn NMR (112 MHz, C_6D_6): δ -491.3.

Grafting of $Sn(OSi(O^tBu)_3)_4$ on SiO_{2-700}: SiO_{2-700} was transferred to a Schlenk flask and suspended in benzene (5 mL). $Sn(OSi(O^tBu)_3)_4$ (1 mmol equiv. with respect to isolated OH groups) was weighed in a separate container and dissolved in benzene (5 mL). The solution of $Sn(OSi(O^tBu)_3)_4$ was then added dropwise to the suspension of SiO_{2-700} while stirring at 100 rpm and benzene was then added until about 1 mm above the SiO_{2-700} slurry. Stirring was continued overnight, after which the liquid phase was decanted, and the material was washed with benzene (2 x 5 mL) and n-pentane (1 x 5 mL). The combined benzene washings were added to a vial containing ferrocene (30 mg, internal standard). An aliquot of (1 mL) combined with benzene-d6 (1 mL) was then analyzed using ^{1}H NMR spectroscopy which allowed for the quantification of remaining precursor and released $HOSi(O^tBu)_3$. The material was dried (10^{-3} mbar for 30 min, then 10^{-5} mbar for 2 hours) affording a white powder.

Elemental analysis results in 2.45 w% Sn (53.6% grafting efficiency, 0.55 Sn/nm^2) in agreement with ^{1}H NMR quantification of released HOSi(O^{t}Bu)$_3$ resulting in 2.64 w% Sn (56.1% grafting efficiency, 0.60 Sn/nm^2). Carbon EA of 2.78 w% C suggests that on average, only one equivalent of OSi(O^{t}Bu)$_3$ ligand remains per grafted Sn atom indicating that the OSi(O^{t}Bu)$_3$ ligands of the grafted precursor are prone to decompose. Indeed, the release of isobutene and tert-butanol is confirmed in the ^{1}H NMR of the washing solution further supporting this decomposition. Grafting on an NMR scale further quantifies the grafting and decomposition process showing that most of the precursor is grafted over the first 12 hours and decomposition to isobutene increases over time (Figure S15).

Elemental Analysis:
2.45 w% Sn, 2.78 w% C, corresponding to 0.55 Sn/nm^2 and a GE of 53.6%

Preparation of SiO_2 supported $Sn^{(IV)}$@SiO_2: The grafted Sn(OSi(O^{t}Bu)$_3$)$_n$/SiO_2 species was placed in a quartz flow reactor under 1 bar SA flux and heated to 600 °C (300 °C/hour), and kept at 600 °C overnight. A white powder was obtained suggesting that no Sn nanoparticles were formed. Full recovery of the OH density of $SiO_{2\text{-}700}$ was assumed.

Elemental Analysis:
2.45 w% Sn corresponding to Sn/nm^2

Preparation of bimetallic PtSn@SiO_2 NPs from $Sn^{(IV)}$@SiO_2: $Sn^{(IV)}$@SiO_2 were transferred to a flat bottomed vial, suspended in benzene (5 mL), and cooled to 10°C with a cold water bath placed under the cooling well of the glovebox. Pt(η^3-allyl)(DIA) (1 mmol equiv. with respect to isolated OH groups) was weighed in a separate container and dissolved in benzene (5 mL). The solution of Pt(η^3-allyl)(DIA) was then added dropwise to the suspension of SiO_{2-700} while stirring at 100 rpm and benzene was then added until about 1 mm above the SiO_{2-700} slurry. The mixture was left to stir for 3 hours, after which the liquid phase was decanted, and the material was washed with benzene (2 x 5 mL) and n-pentane (1 x 5 mL). The combined benzene washings were added to a vial containing ferrocene (30 mg, internal standard). An aliquot of (1 mL) combined with benzene-d_6 (1 mL) was then analyzed using ^{1}H NMR spectroscopy which allowed for the quantification of remaining precursor. The material was dried (10^{-3} mbar for 30 min, then 10^{-5} mbar for 2 hours) affording a yellow powder. Next the grafted material was transferred to a quartz flow reactor under 1 bar H_2 flux and heated to 600 °C (300 °C/hour), and kept at 600 °C overnight. Black particles were obtained suggesting reduction of the metal species. Full recovery of the OH density of $SiO_{2\text{-}700}$ was assumed. Figure S5 shows the sequential FTIR spectra of each of the bimetallic materials.

Elemental Analysis:
$Pt_{0.34}Sn$: 2.09 w% Sn, 1.17 w% Pt, corresponding to 0.47 Sn/nm^2 and 0.16 Pt/nm^2
$Pt_{0.68}Sn$: 2.06 w% Sn, 2.31 w% Pt, corresponding to 0.47 Sn/nm^2 and 0.32 Pt/nm^2
$Pt_{1.17}Sn$: 2.09 w% Sn, 4.05 w% Pt, corresponding to 0.47 Sn/nm^2 and 0.56 Pt/nm^2

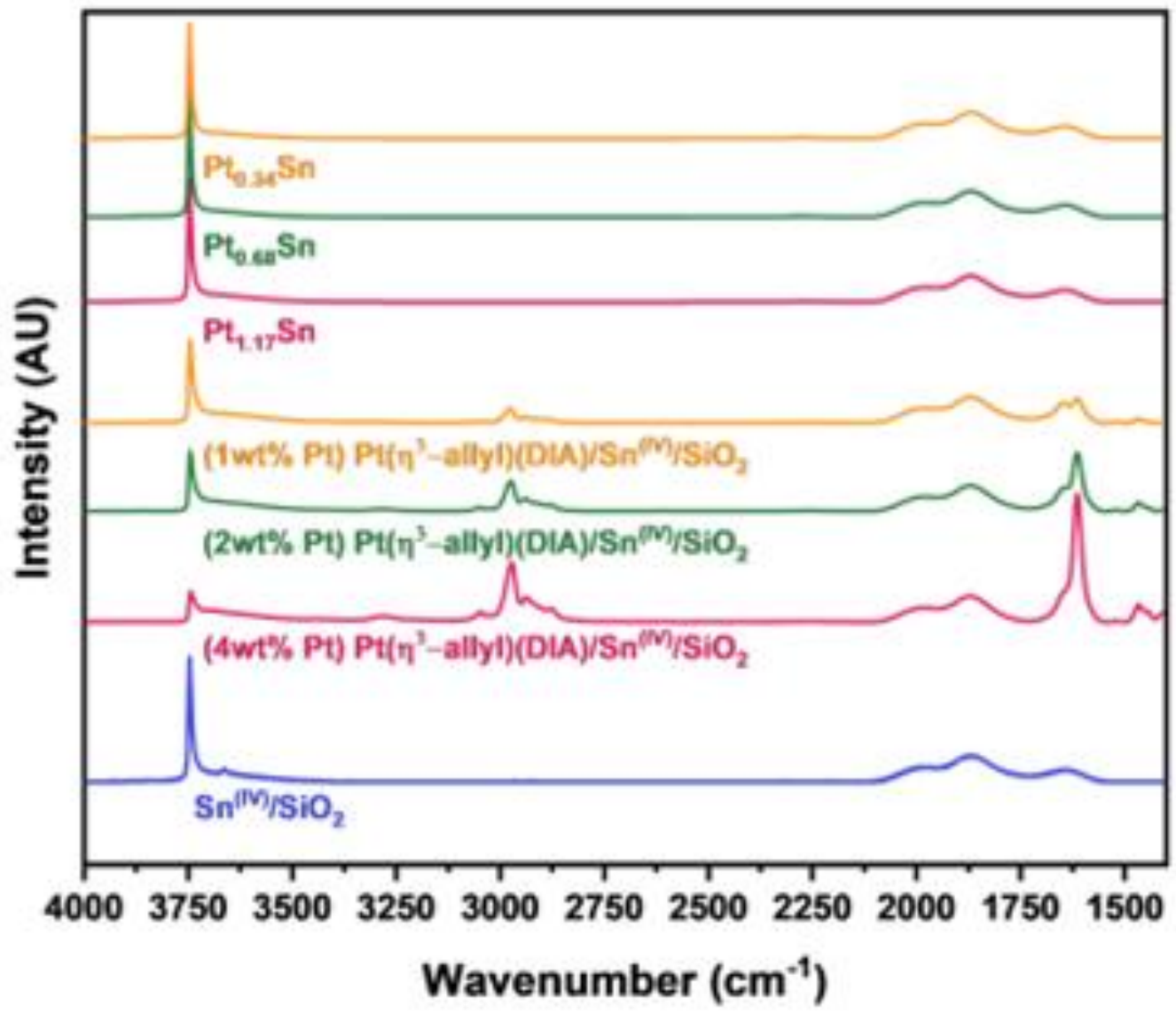

Figure S5. FTIR spectra of grafted and reduced PtSn/SiO_2 materials. High Pt loading (red), medium Pt loading (green) and low Pt loading (yellow).

4 Catalyst Evaluation

4.1 Propane Dehydrogenation Catalytic Tests:

Catalytic tests were carried out utilizing a quartz flow reactor designed in our laboratories and a heating/flow setup designed and produced by Micromeritics Instrument Cooperation (PID Eng & Tech). Catalyst samples were loaded into quartz tubular reactor in an Ar filled glovebox. Prior to exposing the catalyst to flow conditions, a bypass was purged for 20 min with Ar (40 mL/min). The samples were then heated to 550 °C utilizing a tubular furnace under a flow of Ar (40 mL/min) and the temperature was maintained until fully stabilized. The gas mixture containing propane and Ar was subsequently mixed and purged through a bypass for 20 min at the desired flow rate for the reaction prior to contact with the catalyst. Reaction temperatures were maintained utilizing a quartz encased thermocouple maintained in contact with the catalyst dispersed in SiC to yield a total weight of ca. 2.5 g. For a typical catalytic test, 15-18 mg of catalysts diluted with 2.5 g of SiC were used while maintaining identical gas composition and flow rate of 50 mL/min with 1/4 C_3H_8/Ar in ratio. The output gas composition was analyzed automatically by a GC with a flame ionization detector (FID) which was programmed to sample the gas stream every 9 minutes throughout the time of reaction. Gases were purified by passing through a column with molecular sieves and Q5 catalyst prior to introduction to the flow reactor. Propane conversion and selectivity were calculated according to the following equations:

$$\text{Conversion} = X_{C_3H_8} = 1 - \frac{n_{C_3H_8,out}}{n_{C_3H_8,out} + n_{C_3H_6,out} + \frac{n_{C_2H_6,out}}{3/2} + \frac{n_{C_2H_4,out}}{3/2} + \frac{n_{CH_4,out}}{3}}$$

$$\text{Selectivity} = S_i = \frac{n_{i,out} / \frac{3}{c_i}}{n_{C_3H_6,out} + \frac{n_{C_2H_6,out}}{\frac{3}{2}} + \frac{n_{C_2H_4,out}}{\frac{3}{2}} + \frac{n_{CH_4,out}}{3}}$$

(c_i = number of carbon atoms in the respective molecule i)

4.1 Analysis of Catalyst Deactivation

The deactivation rates for the catalysts are calculated using a modified stretched exponential function to fit the forward activity over time. This function assumes that the catalyst is composed of an ensemble of active sites that decay at unique rates.[15] We define the stretched exponential as

$$f(t) = Ae^{\left(-\left(\frac{t}{\tau}\right)^{\beta}\right)}.$$

Where τ is the characteristic time which describes the point at which the system has reached 36.8% of the initial value A, and β is an homogeneity factor between 0 and 1 where β = 1 would imply that each of the sites decay in the exact same process. In addition, our systems exhibit an activation period. We choose to model this activation period with a second stretched exponential this time as a rise. The two functions can then be combined to form a product where initially the process is dominated by the rising stretched exponential, but as the activation period reaches its end, the rise term approaches 1 and the decaying exponential takes over. This is defined as

$$f(t) = A\left(1 - e^{\left(-\left(\frac{t}{\tau_{rise}}\right)^{\beta_{rise}}\right)}\right)\left(e^{\left(-\left(\frac{t}{\tau_{decay}}\right)^{\beta_{decay}}\right)}\right).$$

Here, A is a fitting parameter which fits to a theoretical maximum of the function.

The forward activity is defined as the rate of the forward PDH reaction at the given time point and calculated by:

$$P_f = P_{net}\left(1 - \frac{[C_3H_6][H_2]}{[C_3H_8]K_{eq}}\right)^{-1}.$$

Where P_{net} is the productivity in (mol(C_3H_6)/mol(Pt)/h), [C_3H_6] and [C_3H_8] denote the partial pressures of propene and propane and K_{eq} = 0.09804 is the equilibrium constant of the PDH reaction at 2 bar(g), 10 ml/min C_3H_8 and 40 ml/min Ar flow, and 550°C.

We then perform curve fitting in Python to simulate the forward activity using a stretched exponential. The amplitude (A) is a fitting parameter that must be above the maximum observed activity, τ_{rise}, τ_{decay} ,β_{rise} and β_{decay} are fitted using curve_fit from the SciPy[16] computing library. The quality of the fit can be judged by the standard deviation (σ) and the R^2 of the simulated data from the fit compared to the actual rection data.

The fitting results for the bimetallic catalysts are given in Figure S6 -Figure **S8Error! Reference source not found.**8 and Table S1. We observe that both $Pt_{0.34}Sn$ show higher τ_{decay} values further supporting the superior stability of these catalysts and their associated Pt_1Sn_1 phase in comparison to the less stable $Pt_{1.17}Sn$ catalyst which contains both a Pt_3Sn phase and Pt_1Sn_1 phase.

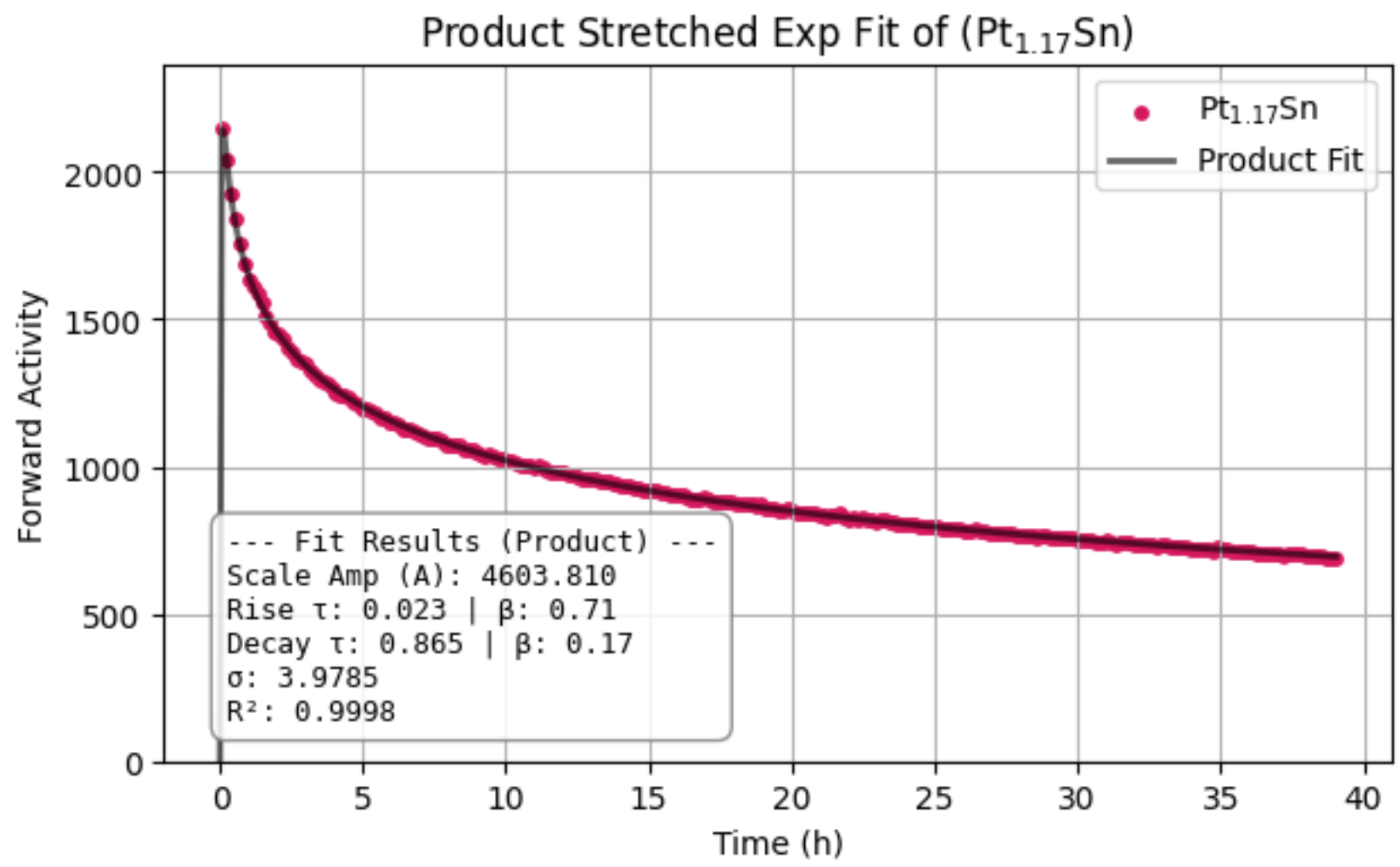

Figure S6. Stretched exponential fitting of forward activity data for $Pt_{1.17}Sn$ from t_0 = 0.08 h.

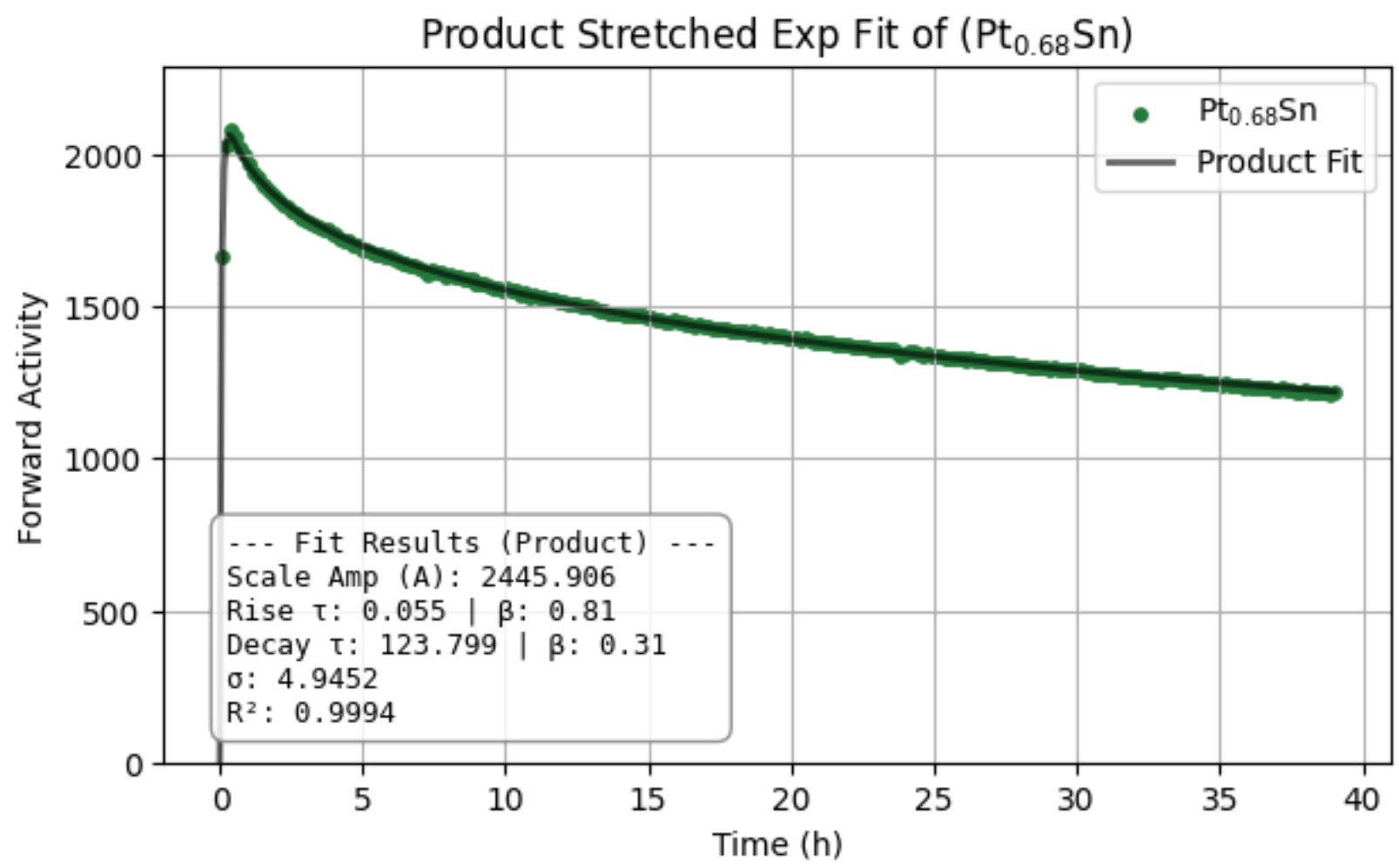

Figure S7. Stretched exponential fitting of forward activity data for $Pt_{0.68}Sn$ from t_0 = 0.08 h.

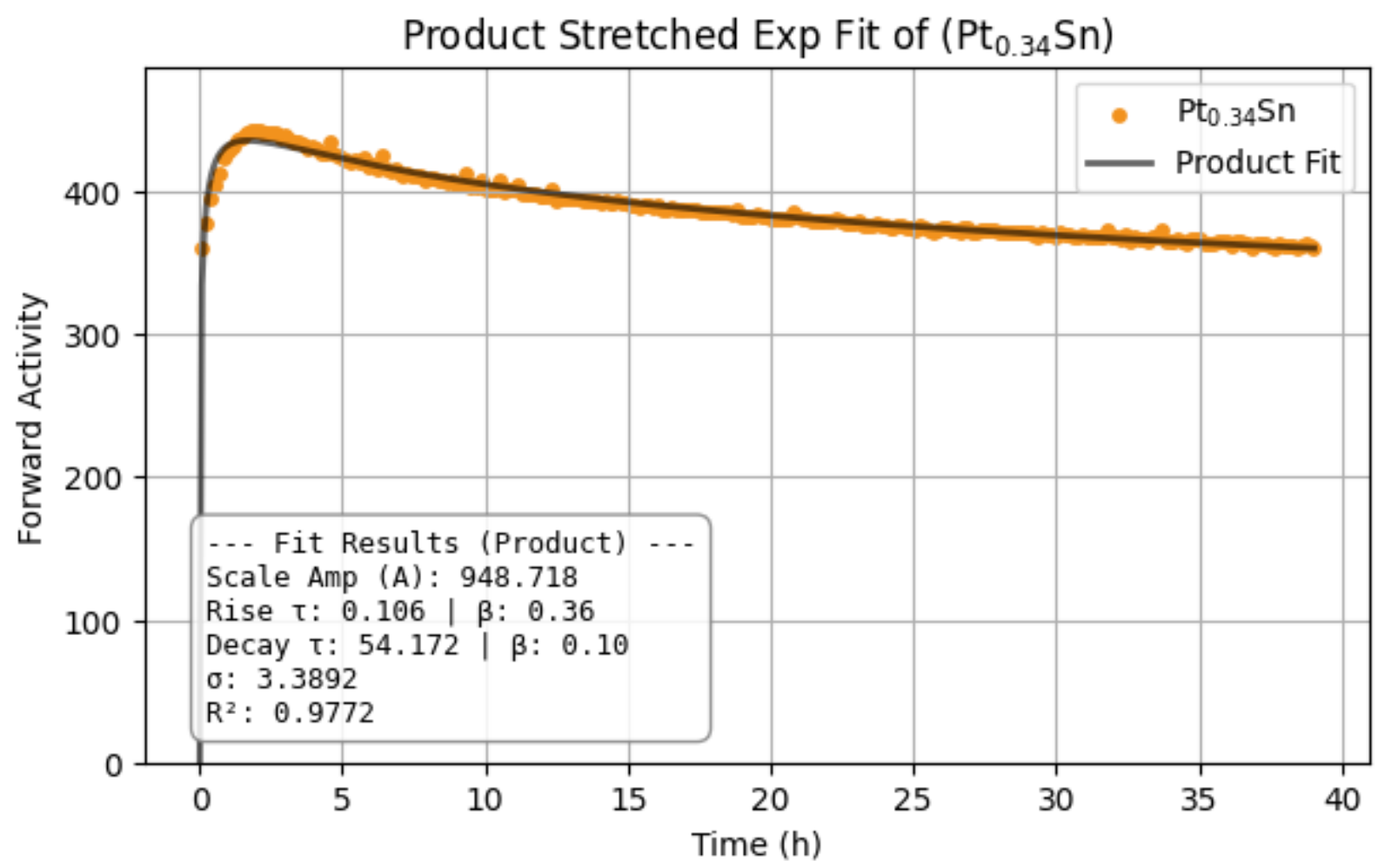

Figure S8. Stretched exponential fitting of forward activity data for $Pt_{0.34}Sn$ from t_0 = 0.08 h.

Table S1. Summary of stretched exponential fitting results with the characteristic time (τ_{rise}, τ_{decay}), homogeneity constant (β_{rise}, β_{decay}), standard deviation of the fit (σ) and R^2 of the fit.

Material	A	τ_{rise} (h)	β_{rise}	τ_{decay} (h)	β_{decay}	σ	R^2
$Pt_{1.17}Sn$	4600	0.023	0.71	0.865	0.17	3.97	0.999
$Pt_{0.68}Sn$	2445	0.055	0.81	123.79	0.31	4.94	0.999
$Pt_{0.34}Sn$	949	0.106	0.36	54.172	0.10	3.38	0.977
Pt	---	---	---	0.005	0.08	4.73	0.94

5 Supporting Figures and Tables

5.1 NMR Spectroscopy:

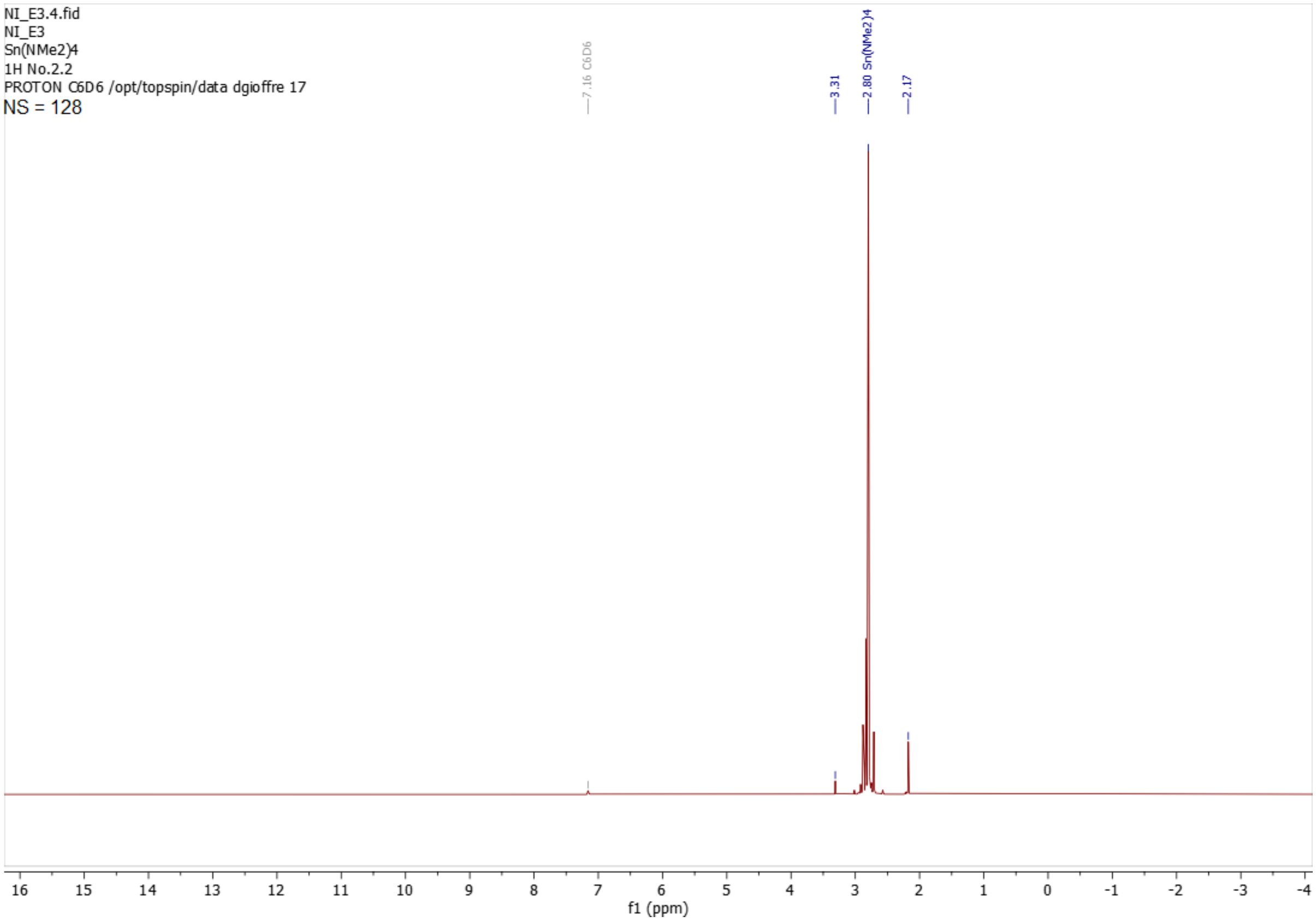

Figure S9. ¹H NMR spectrum of $Sn(NMe_2)_4$. This compound was used without further purification.

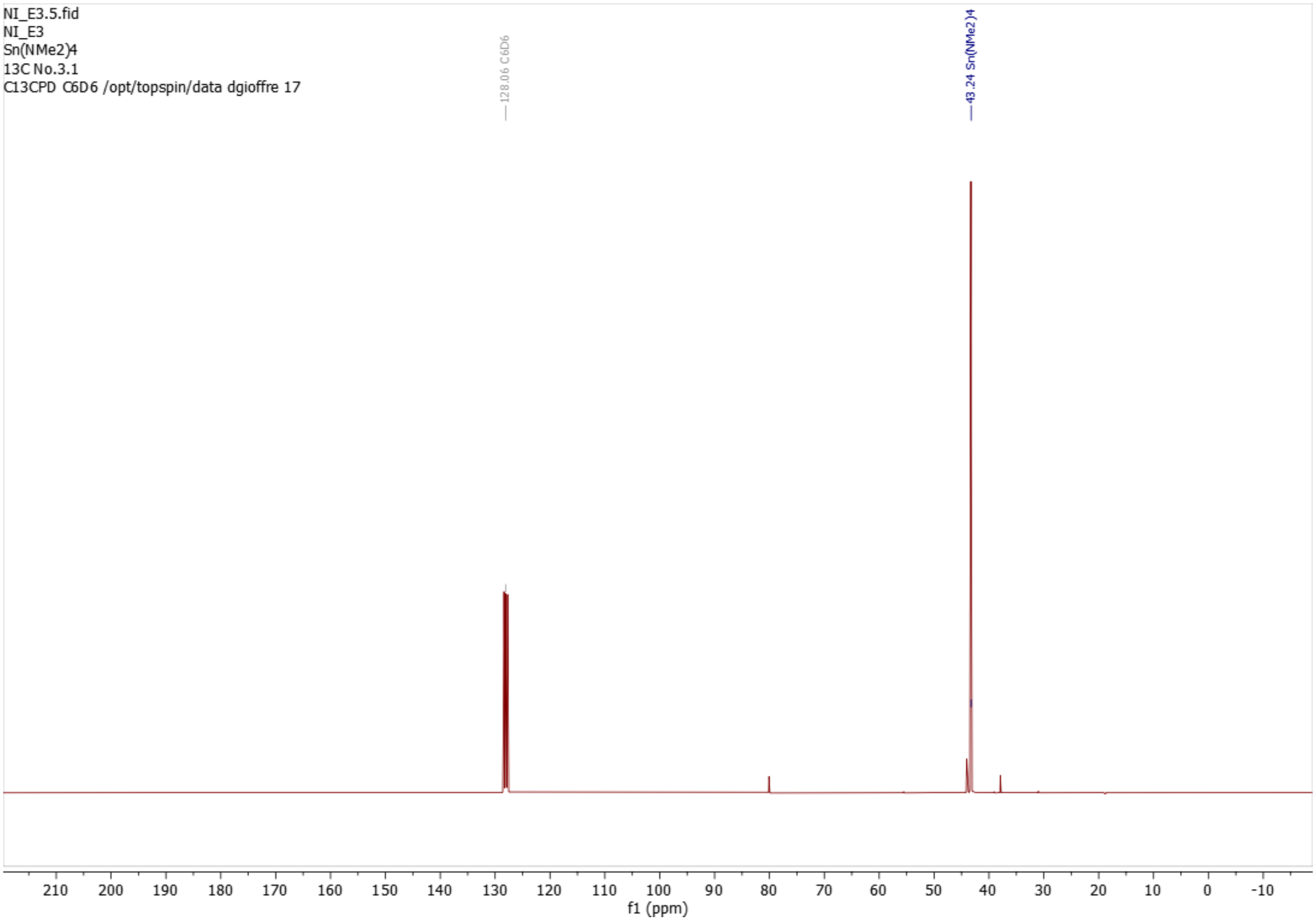

Figure S10. ^{13}C NMR of $Sn(NMe_2)_4$. This compound was used without further purification.

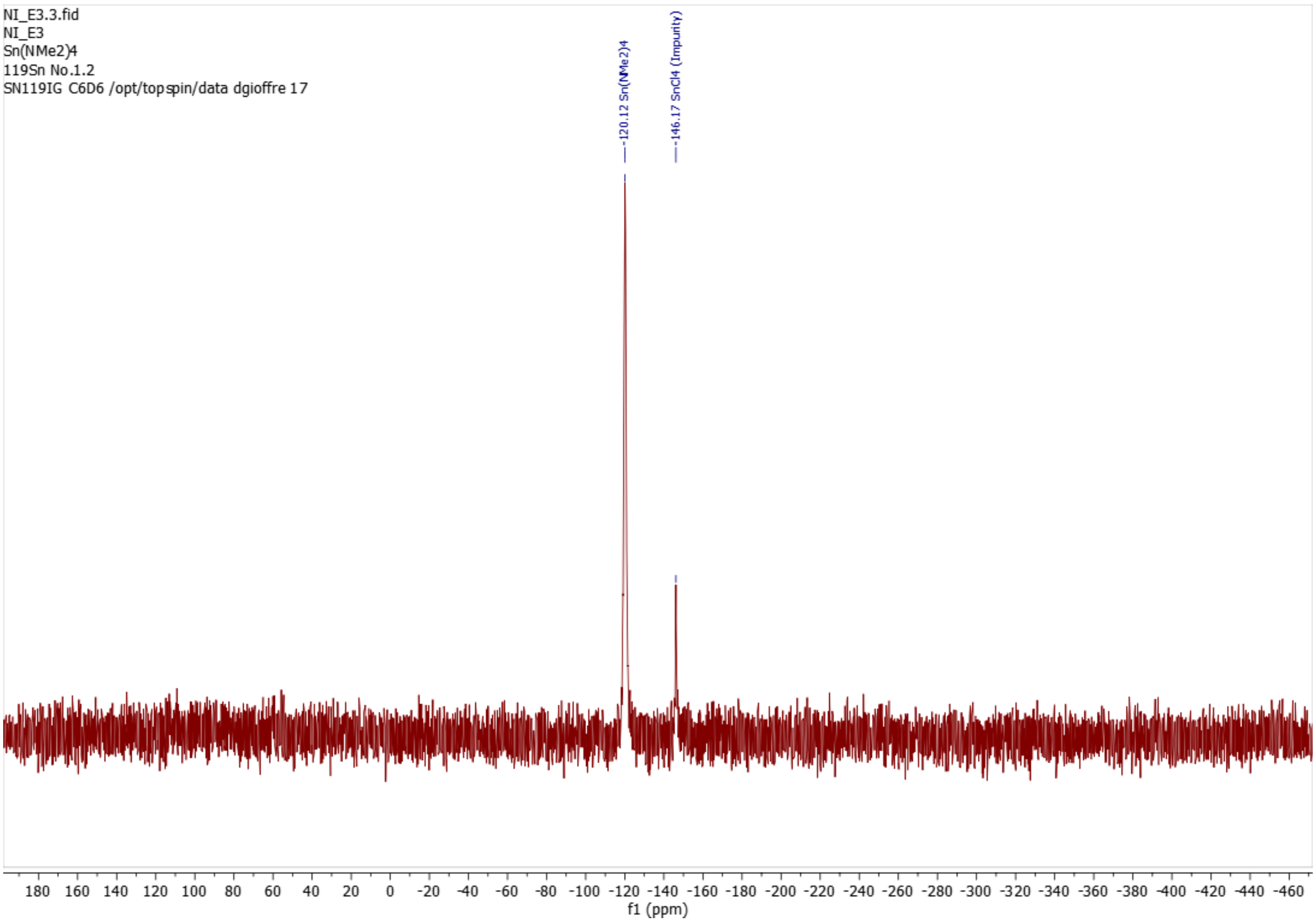

Figure S11. ^{119}Sn NMR of $Sn(NMe_2)_4$. This compound was used without further purification.

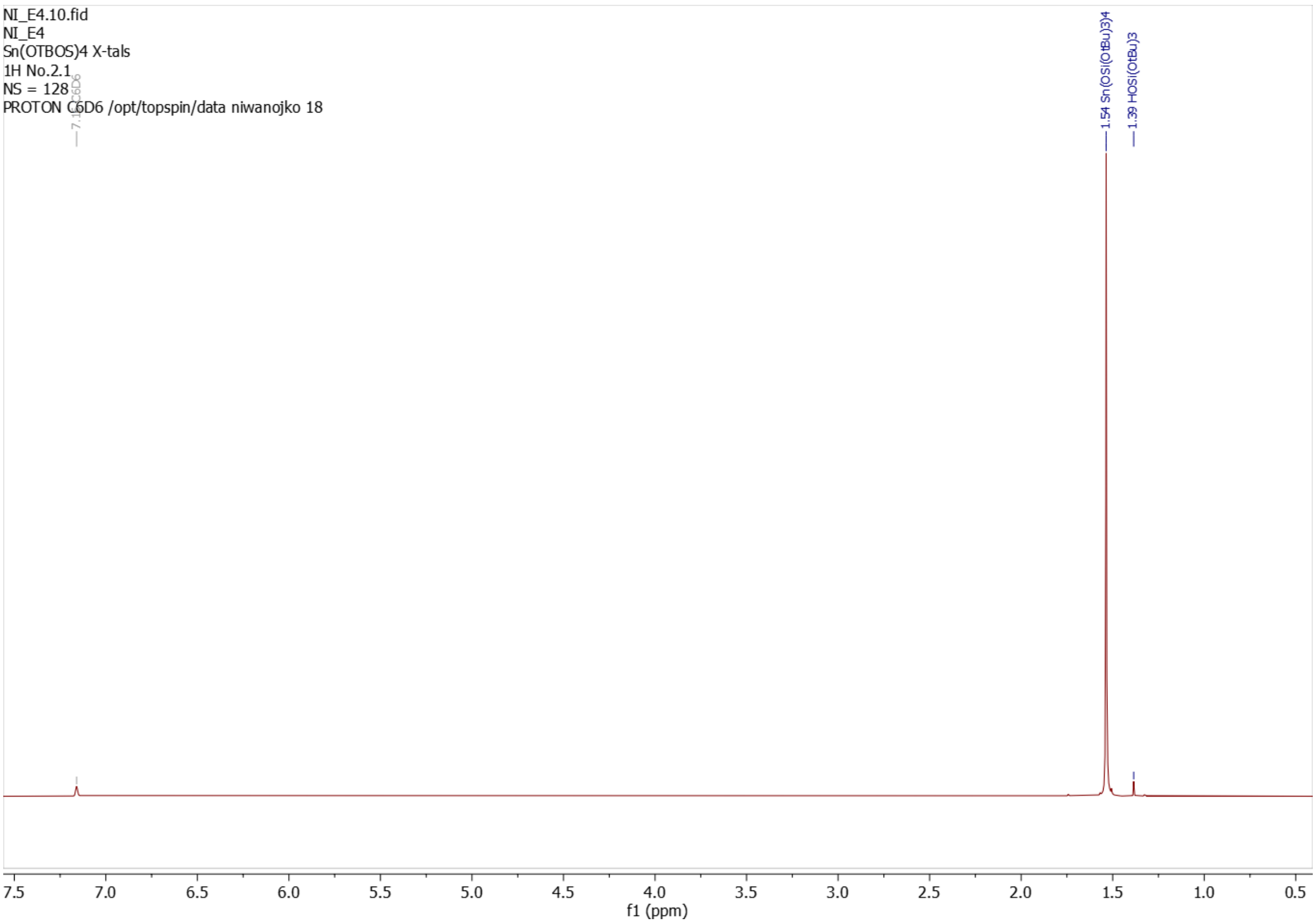

Figure S12. ^{1}H NMR of $Sn(OSi(O^tBu)_3)_4$.

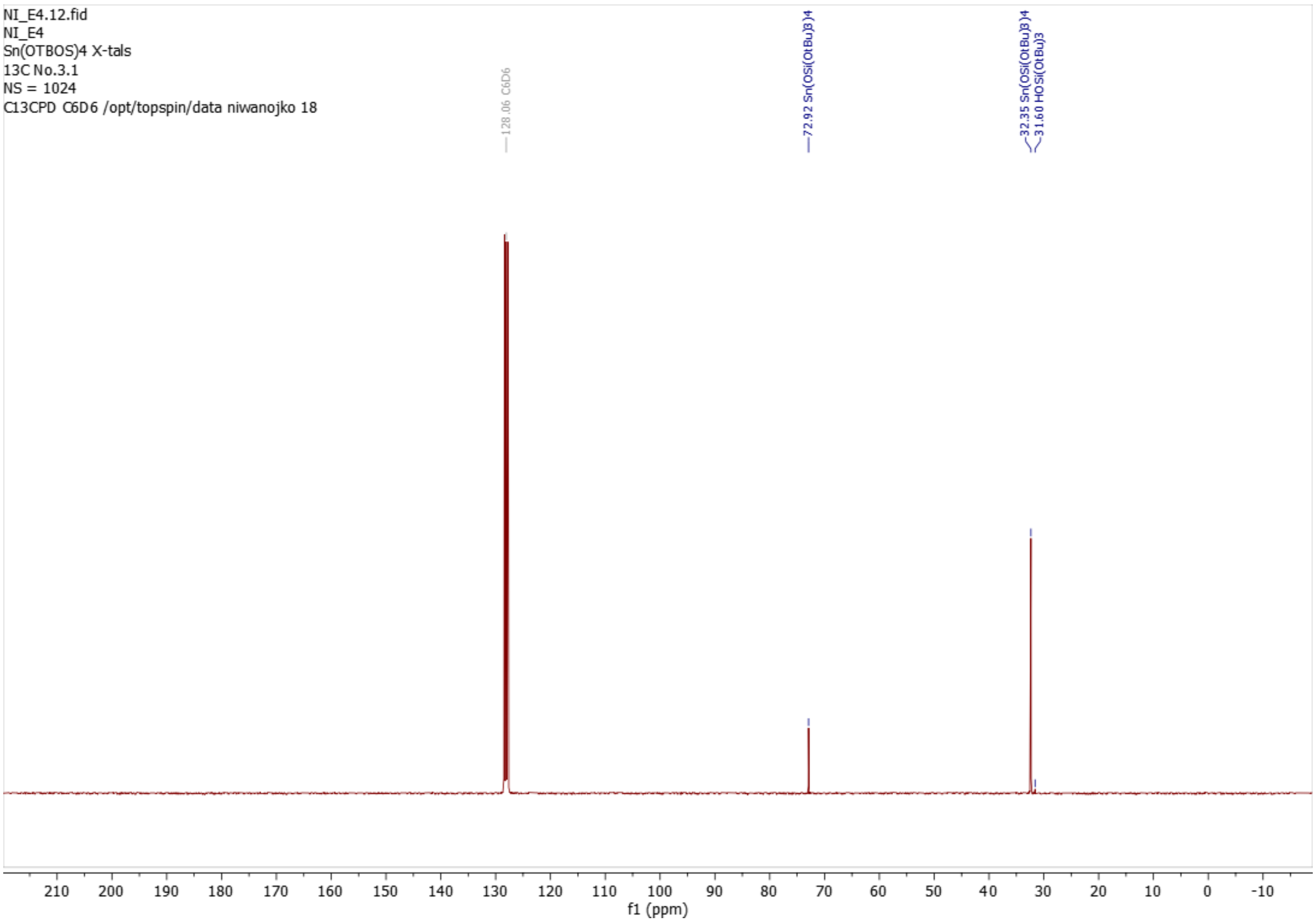

Figure S13. ^{13}C NMR of $Sn(OSi(O^tBu)_3)_4$.

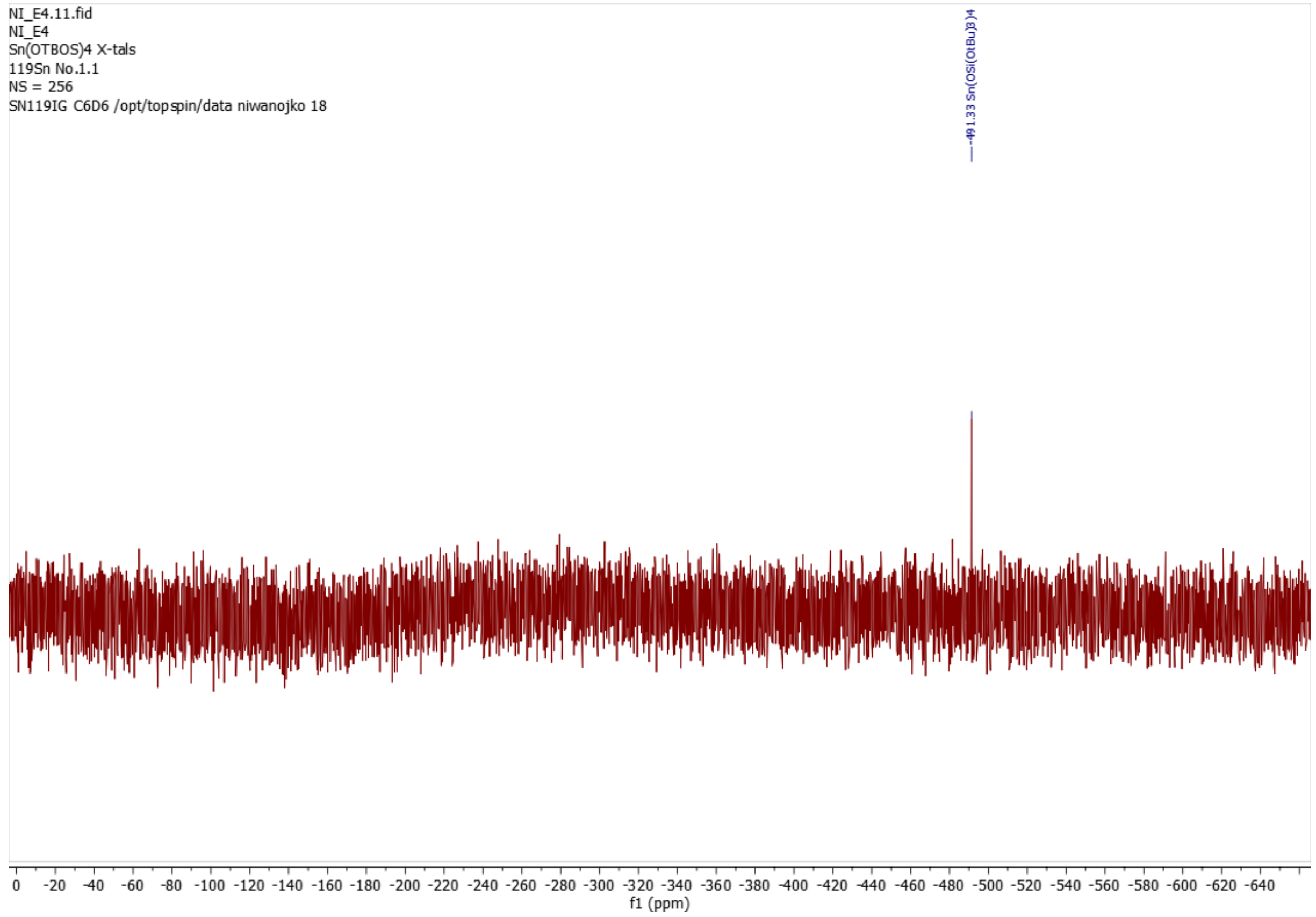

Figure S14. ^{119}Sn NMR of Sn(OSi(O^{t}Bu)$_3$)$_4$.

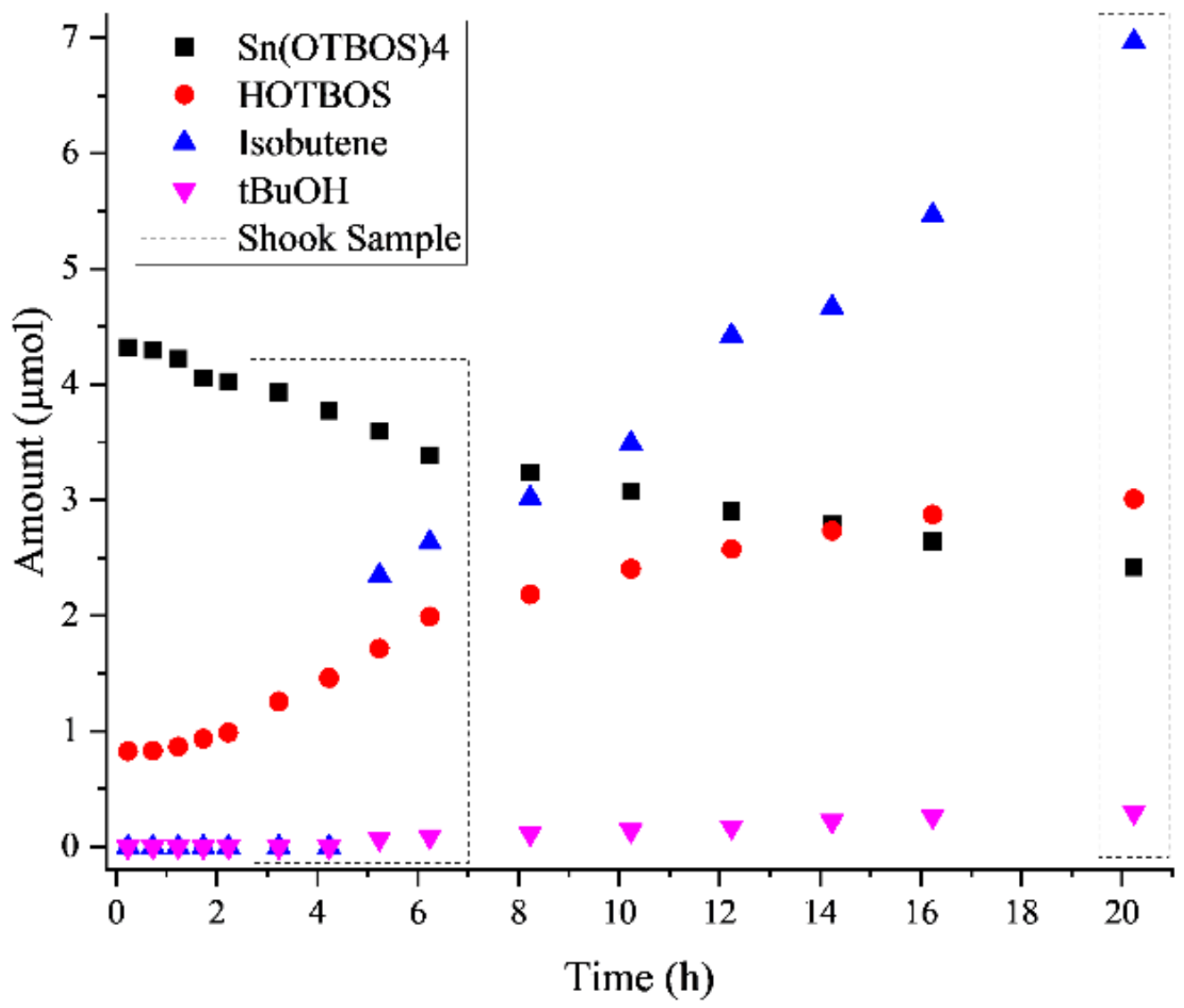

Figure S15. NMR scale grafting of Sn(OSi(O^{t}Bu)$_3$)$_4$. Note that the amount of isobutene released cannot be correctly quantified as it is released as a gas.

5.2 Chemisorption Analysis

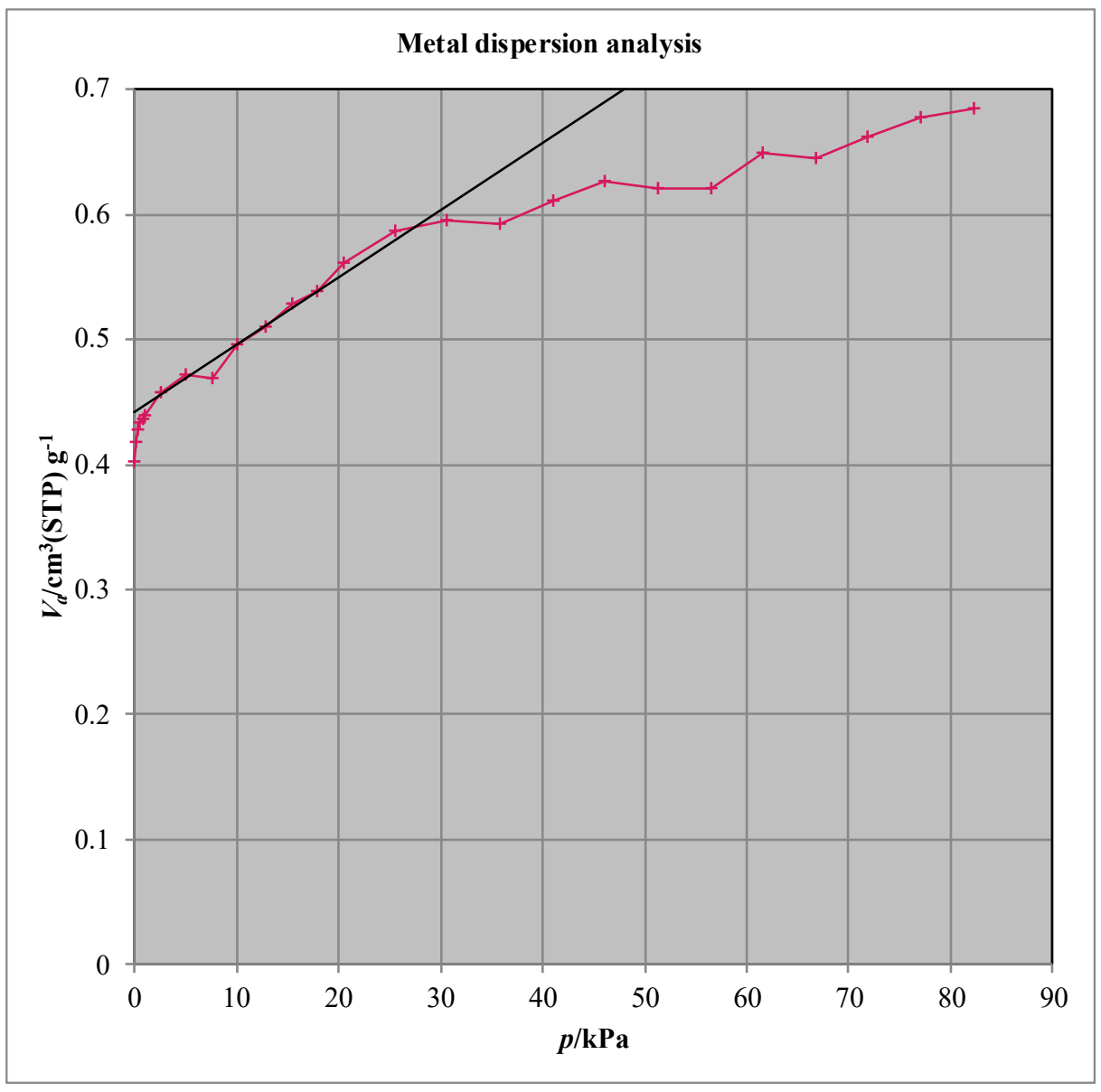

Figure S16. Metal dispersion analysis for CO chemisorption measurements of $Pt_{1.17}Sn$ resulting in a chemisorption volume of 0.4417 cm^3/g and 18.05 µmol $Pt_{Exposed}/g_{cat}$.

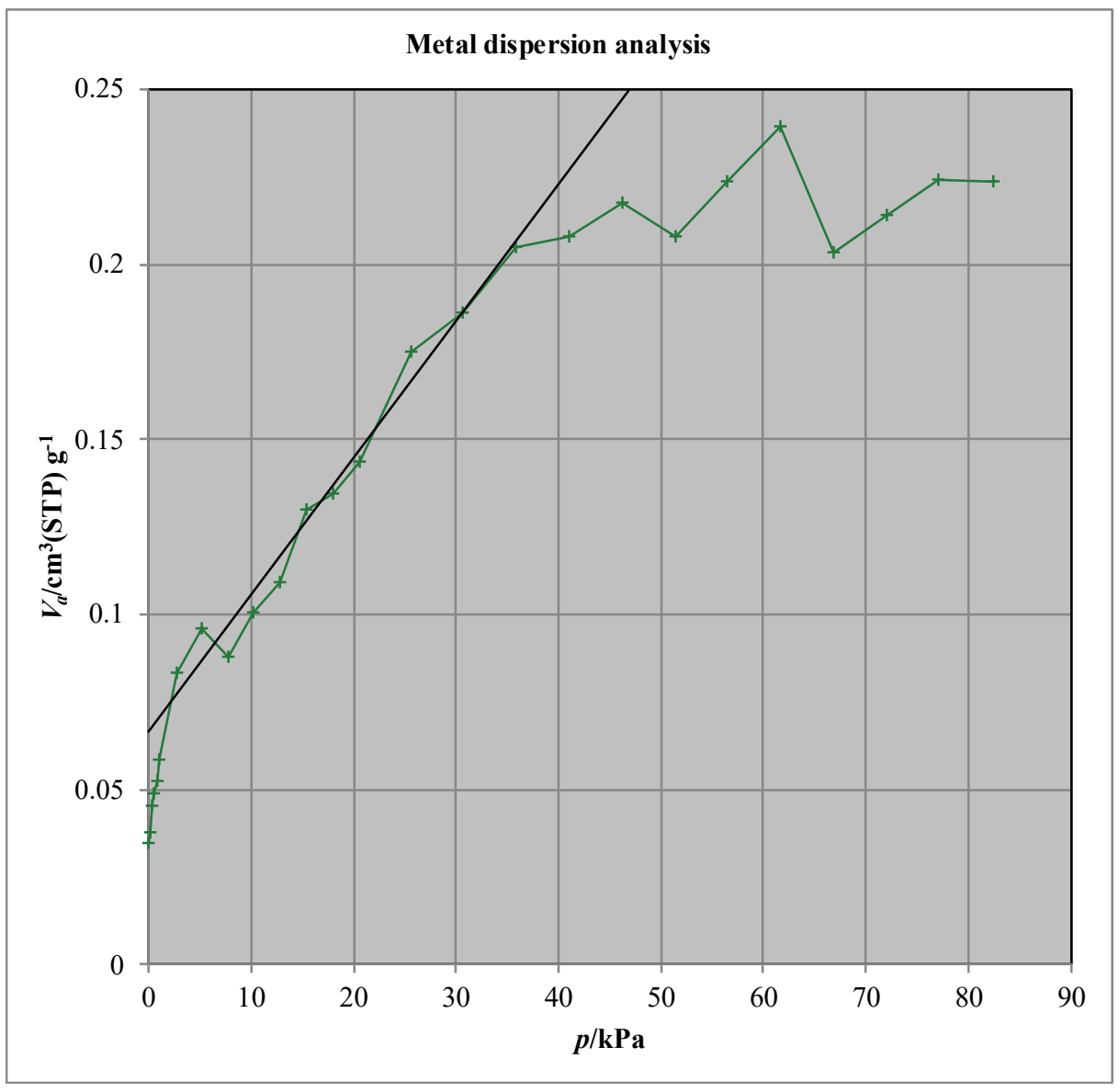

Figure S17. Metal dispersion analysis for CO chemisorption measurements of $Pt_{0.68}Sn$ resulting in a chemisorption volume of 0.0665 cm^3/g and 2.72 µmol $Pt_{Exposed}/g_{cat}$.

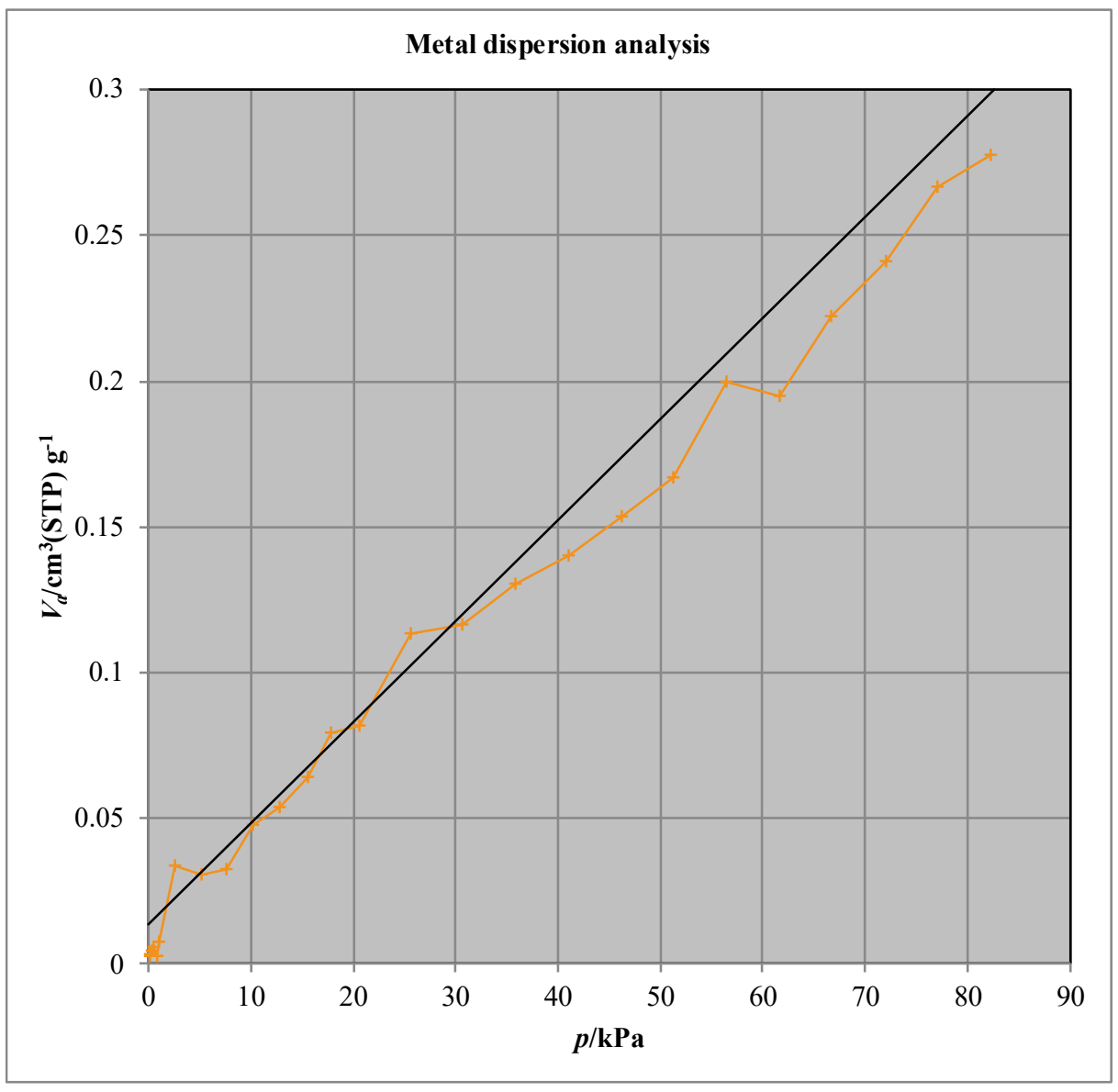

Figure S18. Metal dispersion analysis for CO chemisorption measurements of $Pt_{0.34}Sn$ resulting in a chemisorption volume of 0.0139 cm^3/g and 0.57 µmol $Pt_{Exposed}/g_{cat}$.

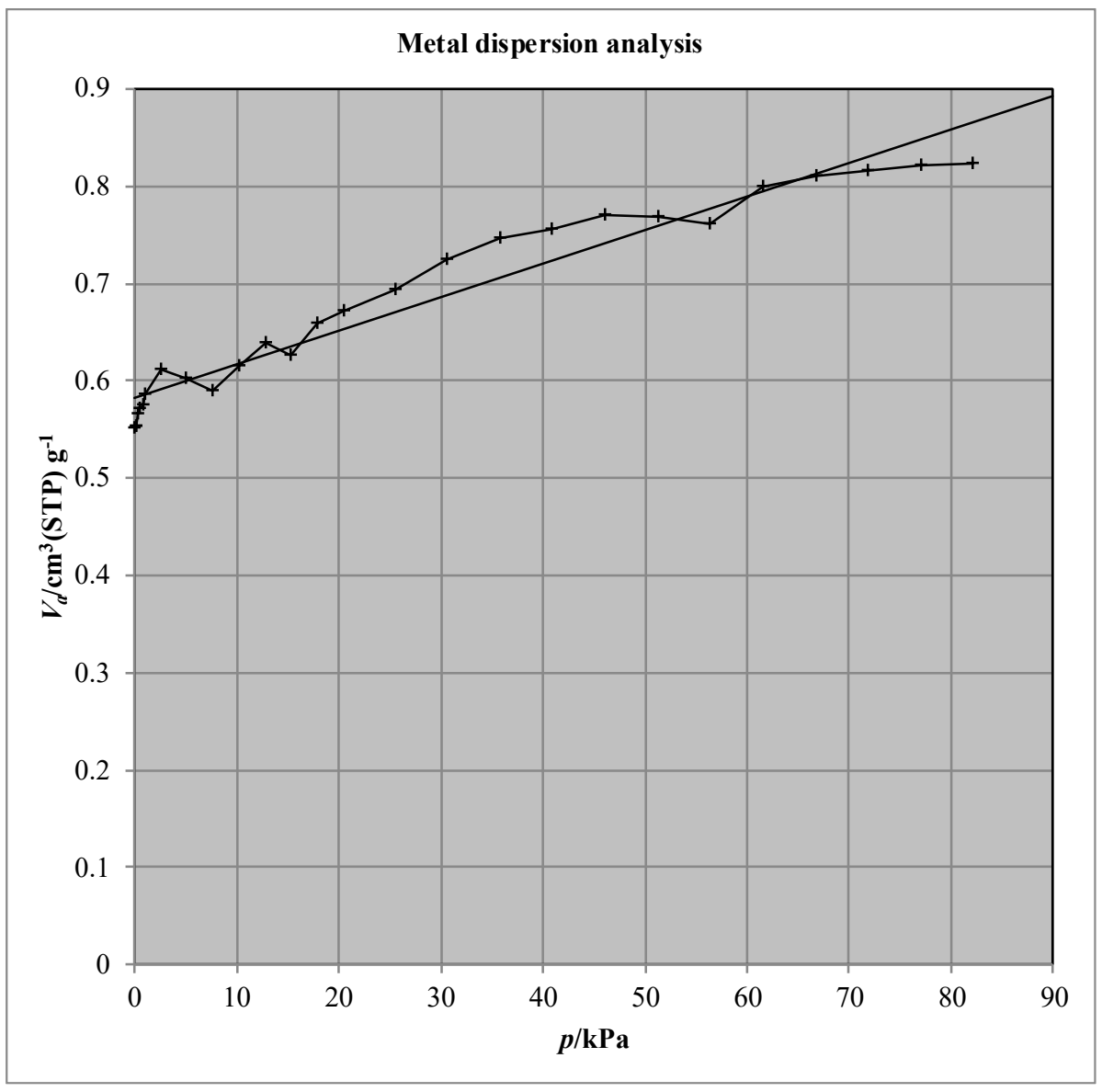

Figure S19. Metal dispersion analysis for CO chemisorption measurements of Pt/SiO_2 resulting in a chemisorption volume of 0.5817 cm^3/g and 23.77 µmol $Pt_{Exposed}/g_{cat}$.

5.3 Electron Microscopy:

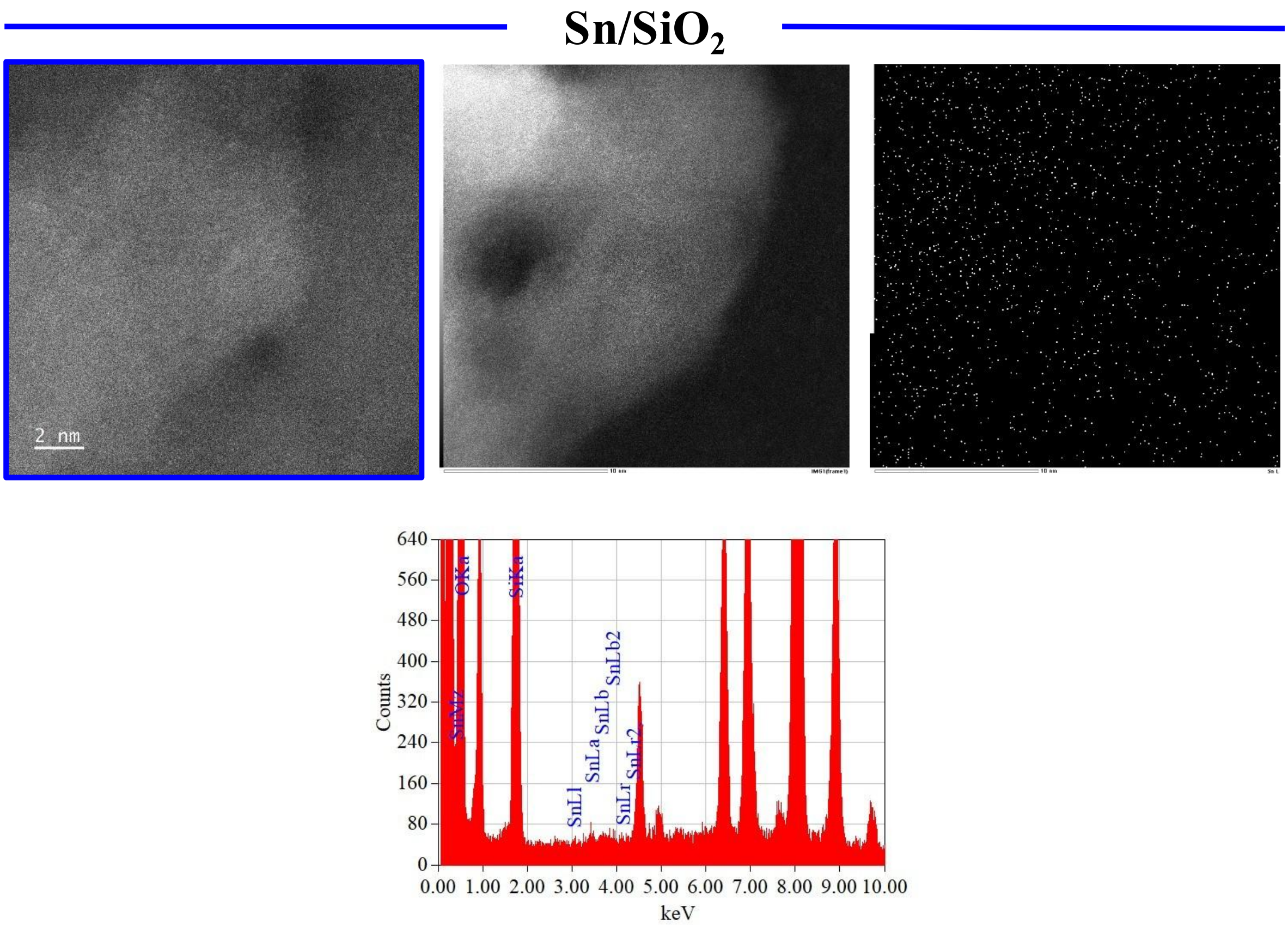

Figure S20. High-resolution HAADF STEM images (left and middle), Sn L EDXS map (right) and spectrum (bottom) of Sn/SiO_2 indicating well dispersed distribution of Sn.

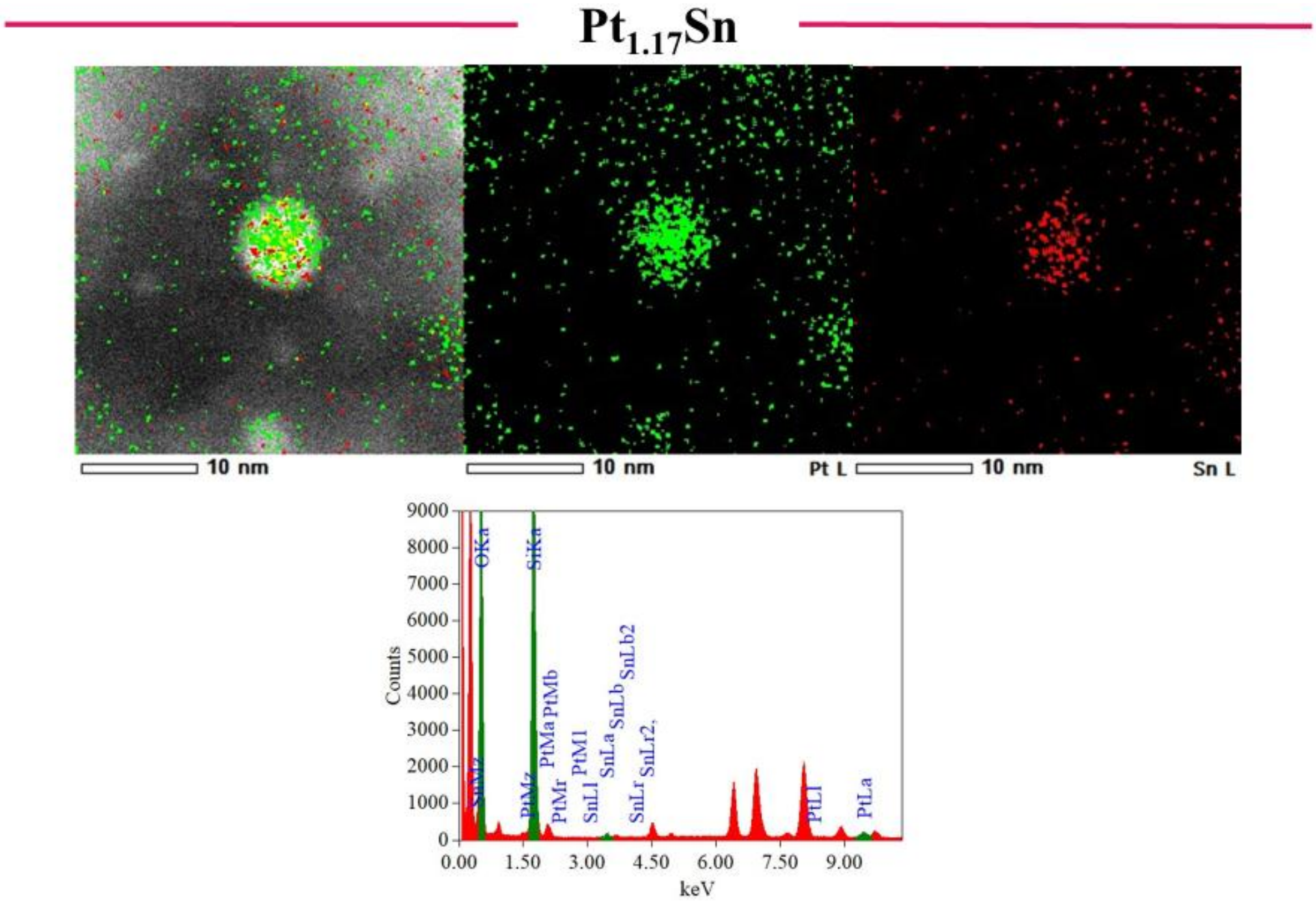

Figure S21. HAADF STEM image and Pt L (green) and Sn L (red) STEM EDXS maps and spectrum of $Pt_{1.17}Sn/SiO_2$ indicating alloying of Pt and Sn within the nanoparticles.

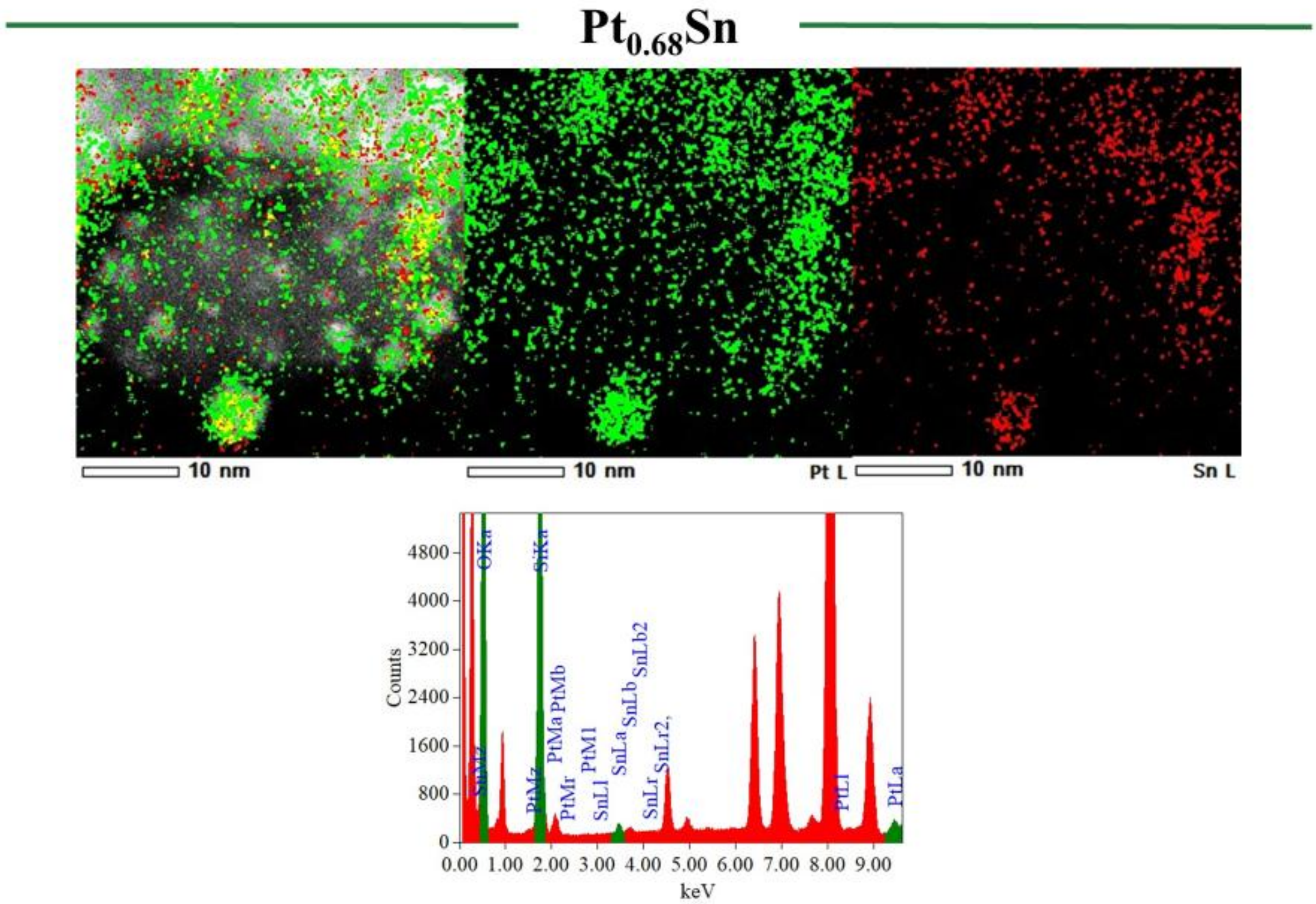

Figure S22. HAADF STEM image and Pt L (green) and Sn L (red) STEM EDXS maps and spectrum of $Pt_{0.68}Sn/SiO_2$ indicating alloying of Pt and Sn within the nanoparticles.

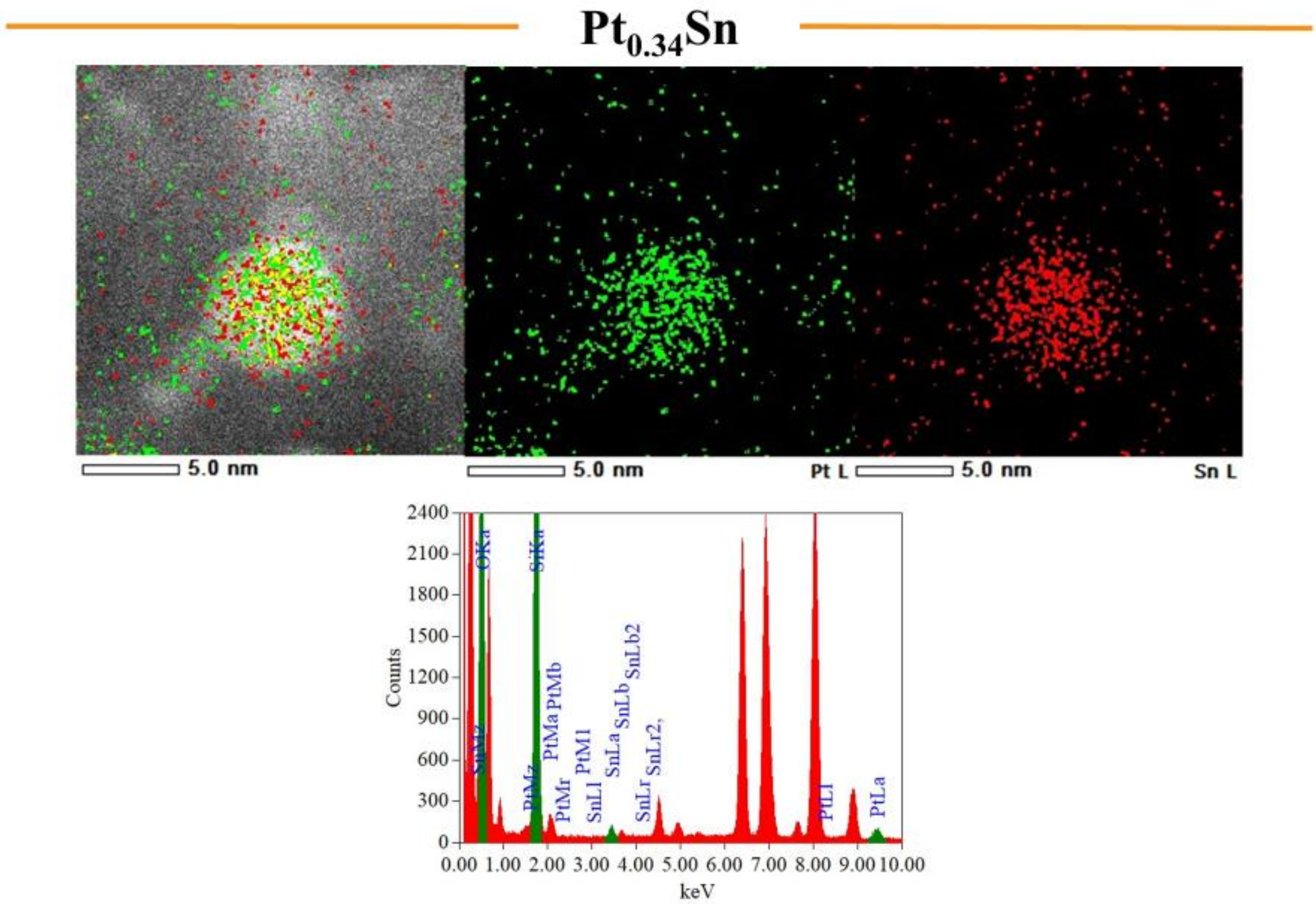

Figure S23. HAADF STEM image and Pt L (green) and Sn L (red) STEM EDXS maps and spectrum of $Pt_{0.34}Sn/SiO_2$ indicating alloying of Pt and Sn within the nanoparticles.

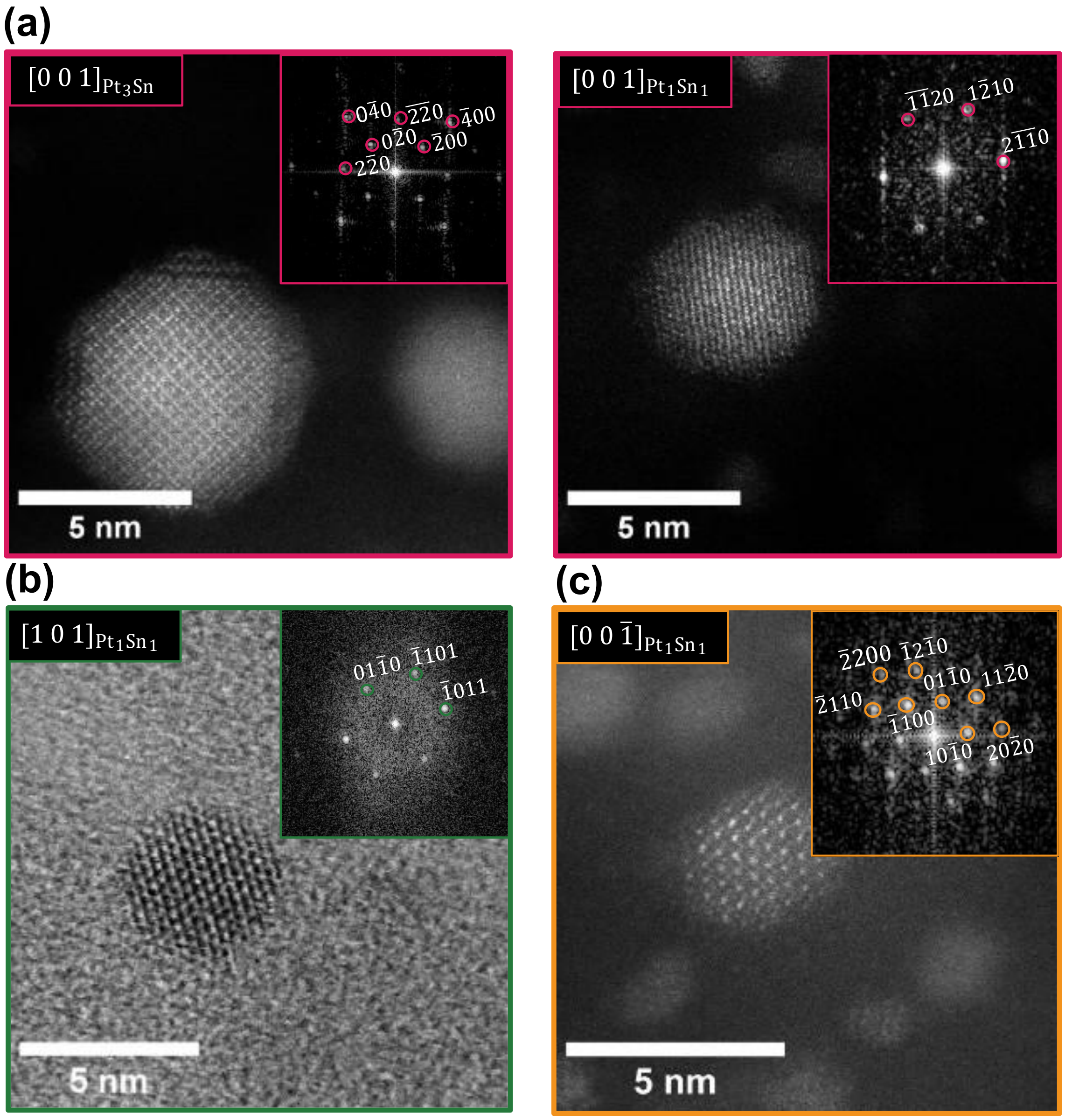

Figure S24. High-resolution HAADF STEM images along with the FFT of crystalline nanoparticles of $Pt_{1.17}Sn$ **(a)**, $Pt_{0.68}Sn$ **(b)** and $Pt_{0.34}Sn$ **(c)** exhibiting both Pt_3Sn_1 and Pt_1Sn_1 phases **(a)** or only a Pt_1Sn_1 phase **(b)**, **(c)**. The FFT inlay is matched to the referenced alloy phase and reported as $[U\ V\ W]_{Pt_xSn_y}$.

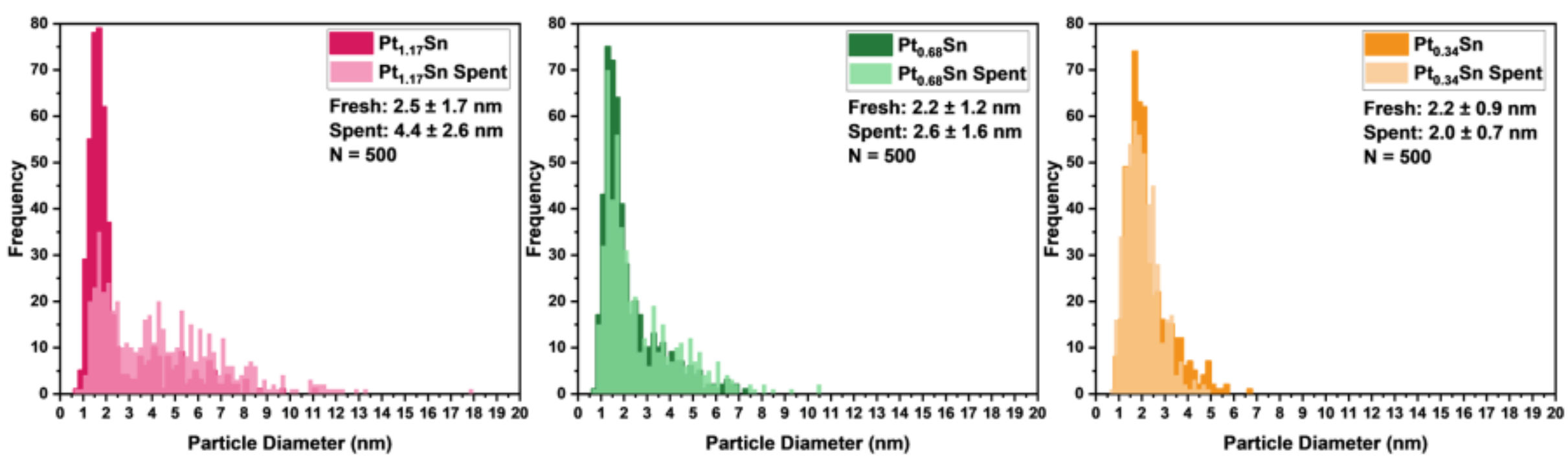

Figure S25. Particle size distribution of the spent PtSn catalysts. Only $Pt_{1.17}Sn$ shows observable sintering after 30 h TOS.

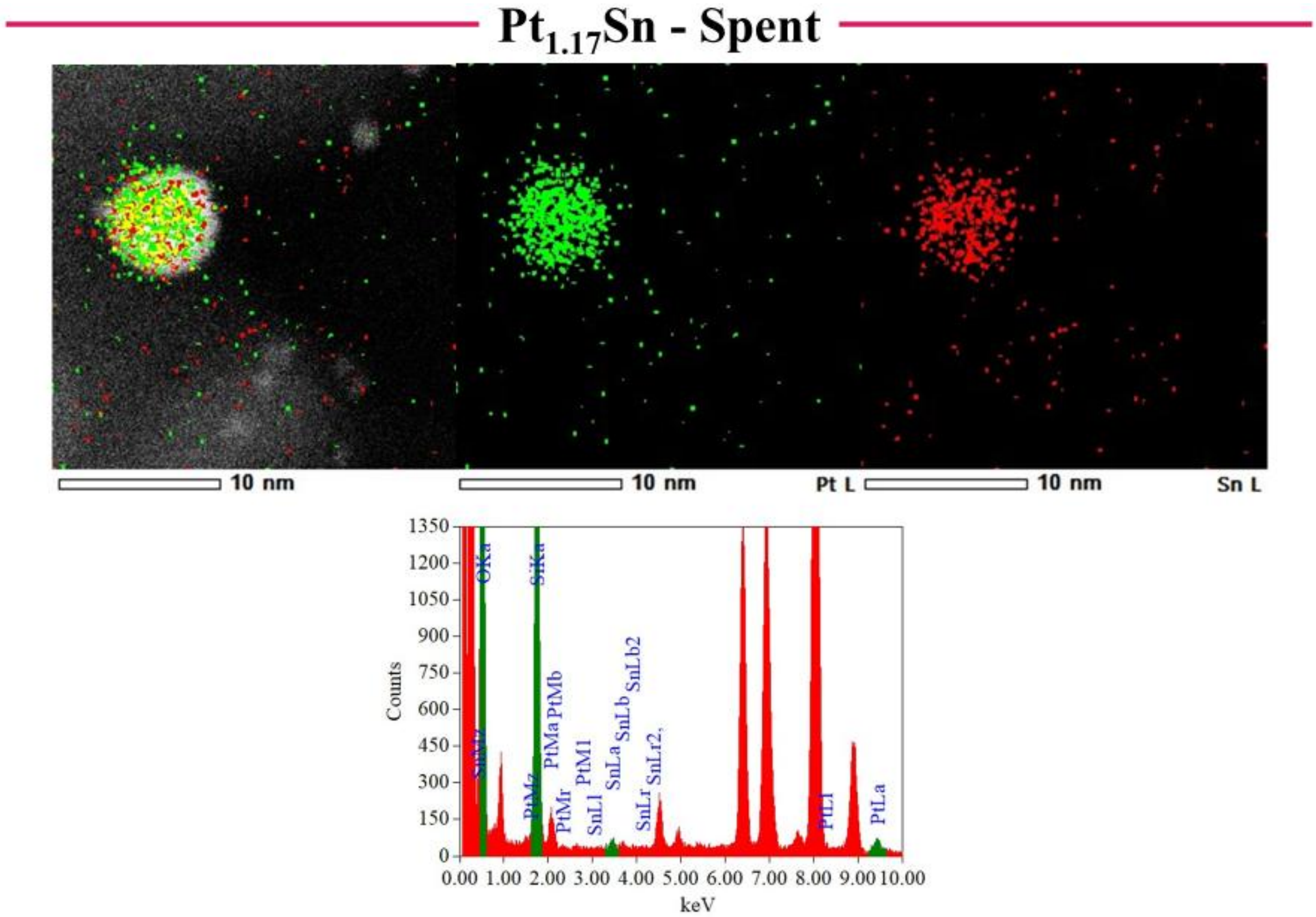

Figure S26. HAADF STEM image and Pt L (green) and Sn L (red) STEM EDXS maps and spectrum of spent $Pt_{1.17}Sn/SiO_2$ after 30 h TOS indicating alloying of Pt and Sn within the nanoparticles.

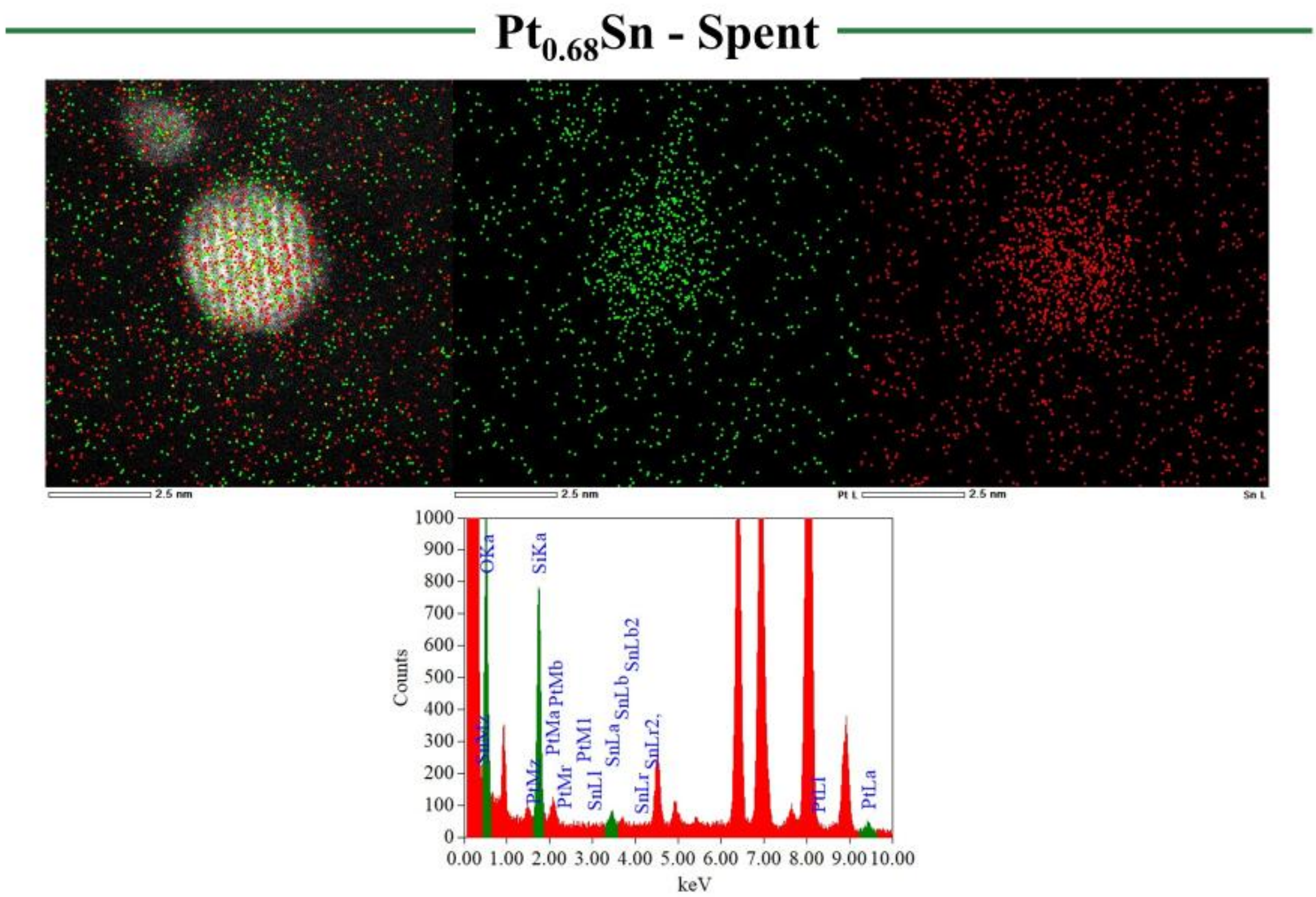

Figure S27. HAADF STEM image and Pt L (green) and Sn L (red) and STEM EDXS spectrum and maps of spent $Pt_{0.68}Sn/SiO_2$ after 30 h TOS indicating alloying of Pt and Sn within the nanoparticles.

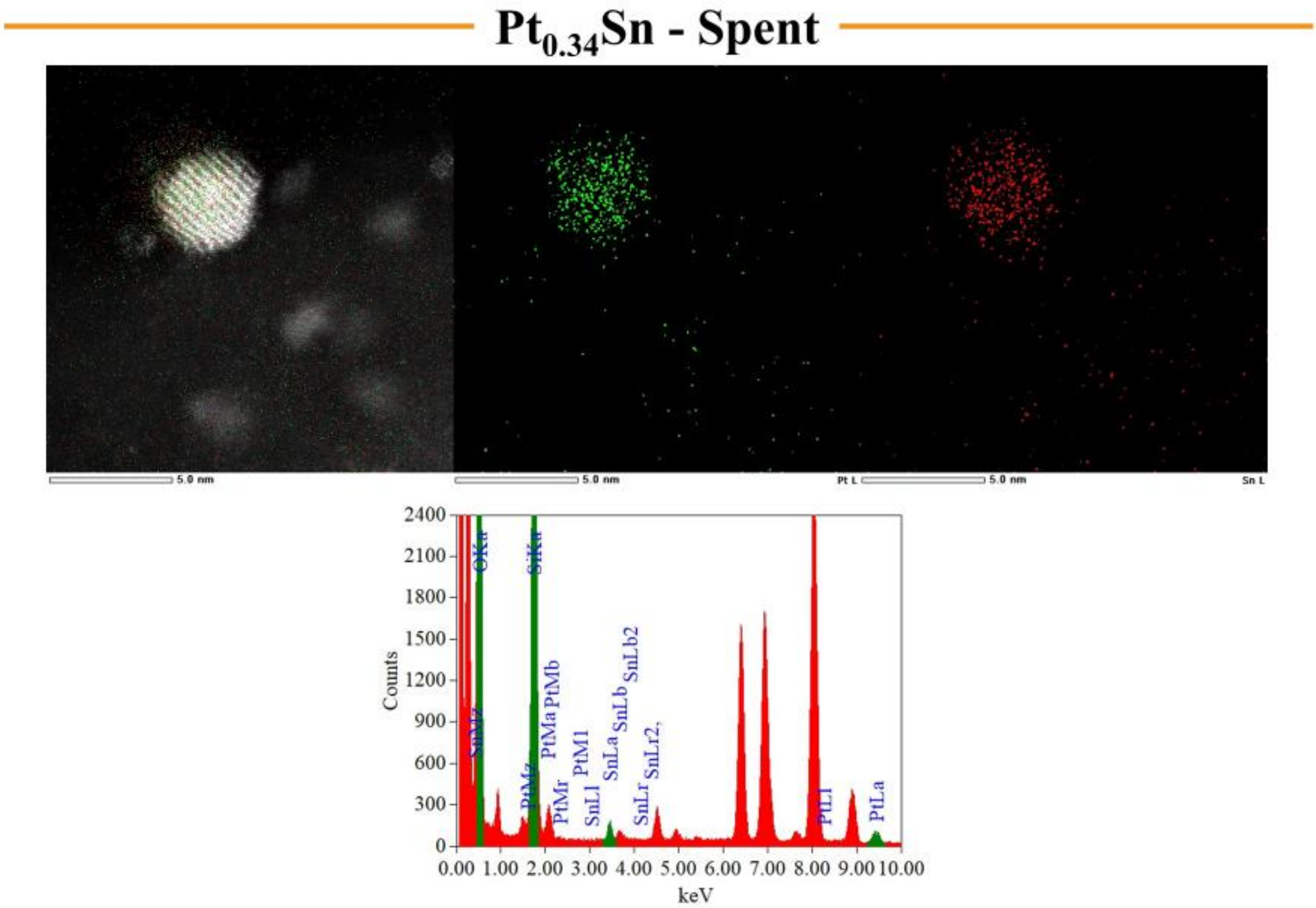

Figure S28. HAADF STEM image and Pt L (green) and Sn L (red) STEM EDXS maps and spectrum of spent $Pt_{0.34}Sn/SiO_2$ after 30 h TOS indicating alloying of Pt and Sn within the nanoparticles.

Table S2. STEM EDXS quantification results of the pre-catalysts from the Pt-L and Sn-L lines performed on the measurements from Figure S211, Figure S222, and Figure S233 for $Pt_{1.17}Sn$, $Pt_{0.68}Sn$ and $Pt_{0.34}Sn$ respectively.

Material	Pt Mass % from Pt-L EDXS	Sn Mass % from Sn-L EDXS	Pt:Sn Ratio from EDXS	Pt:Sn Ratio from Elemental Analysis	Alloys Detected in Sample
$Pt_{1.17}Sn$	0.47	0.33	1.42	1.17	Pt_3Sn and Pt_1Sn_1
$Pt_{0.68}Sn$	0.68	0.50	1.36	0.68	Pt_1Sn_1
$Pt_{0.34}Sn$	0.49	0.51	0.96	0.34	Pt_1Sn_1

Table S3. STEM EDXS quantification results of the post-catalysts from the Pt-L and Sn-L lines performed on the measurements from Figure S266, Figure S277, and Figure S288 for $Pt_{1.17}Sn$, $Pt_{0.68}Sn$ and $Pt_{0.34}Sn$ respectively.

Material	Pt Mass % from Pt-L EDXS	Sn Mass % from Sn-L EDXS	Pt:Sn Ratio from EDXS	Pt:Sn Ratio from Elemental Analysis	Alloys Detected in Sample
$Pt_{1.17}Sn$ - Post	0.52	0.30	1.73	1.17	Pt_3Sn and Pt_1Sn_1
$Pt_{0.68}Sn$ - Post	1.43	1.61	0.89	0.68	Pt_1Sn_1
$Pt_{0.34}Sn$ - Post	0.51	0.68	0.75	0.34	Pt_1Sn_1

Notably, the EDXS quantification does not perfectly match the observed phases form synchrotron XRD. This is likely due to the low signal to noise ratio of the spectrum, which is a result of the short acquisition time due to the occurrence of charging effects, as commonly the case for oxide supported nanoparticles.

5.4 X-Ray Absorption Spectroscopy:

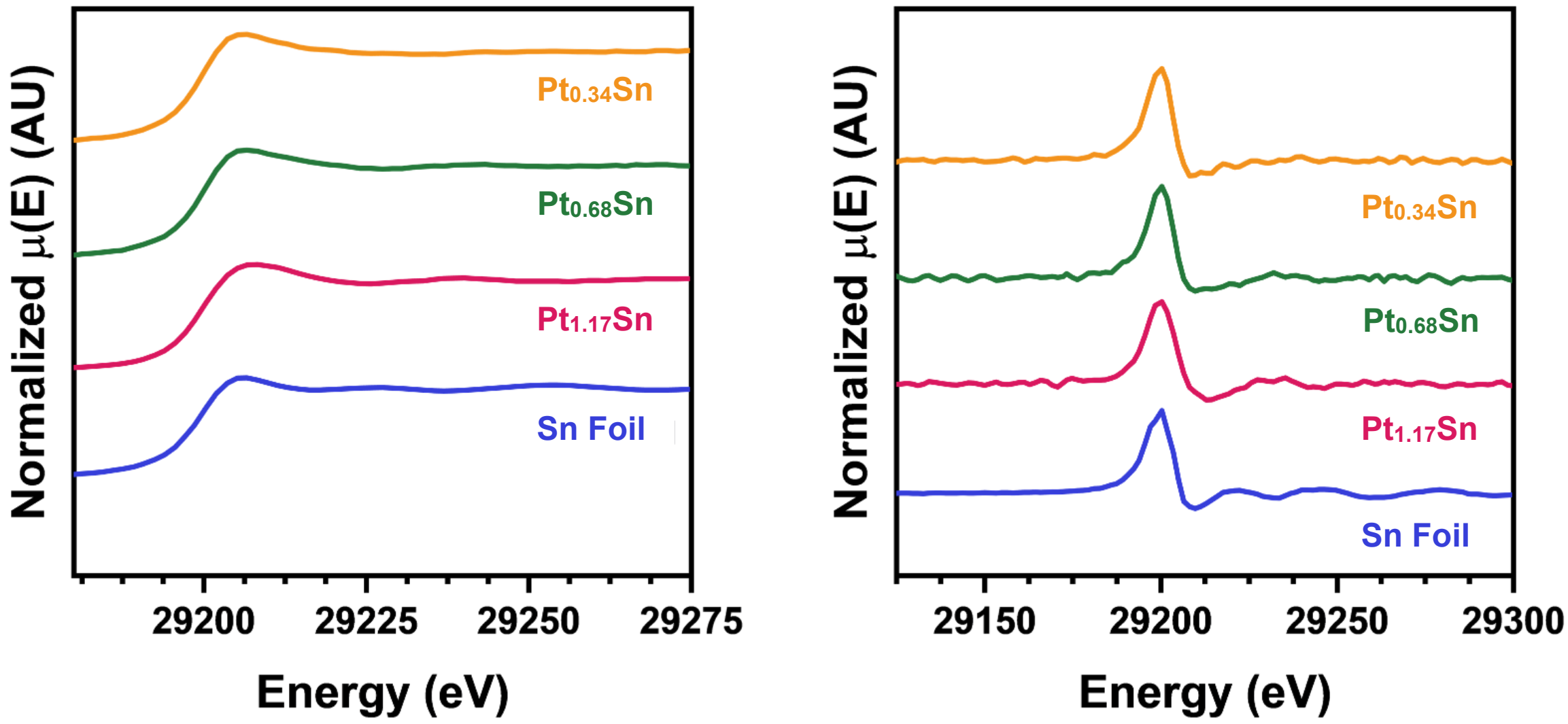

Figure S29. Sn-K edge XANES spectra and their derivative for bimetallic PtSn materials showing that all detected Sn species are in the metallic state.

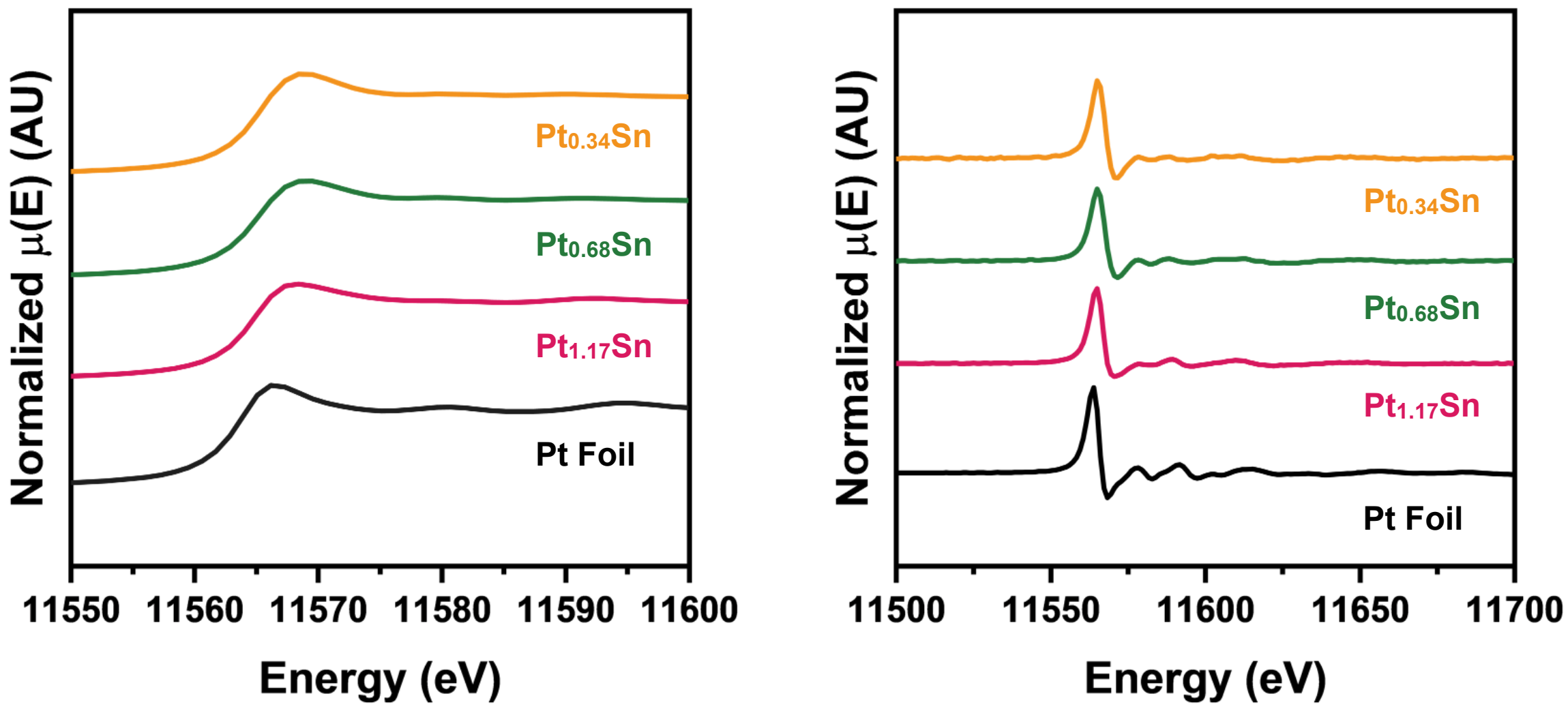

Figure S30. Pt-L_3 edge XANES spectra and their derivative for bimetallic PtSn materials showing that all detected Pt species are in the metallic state.

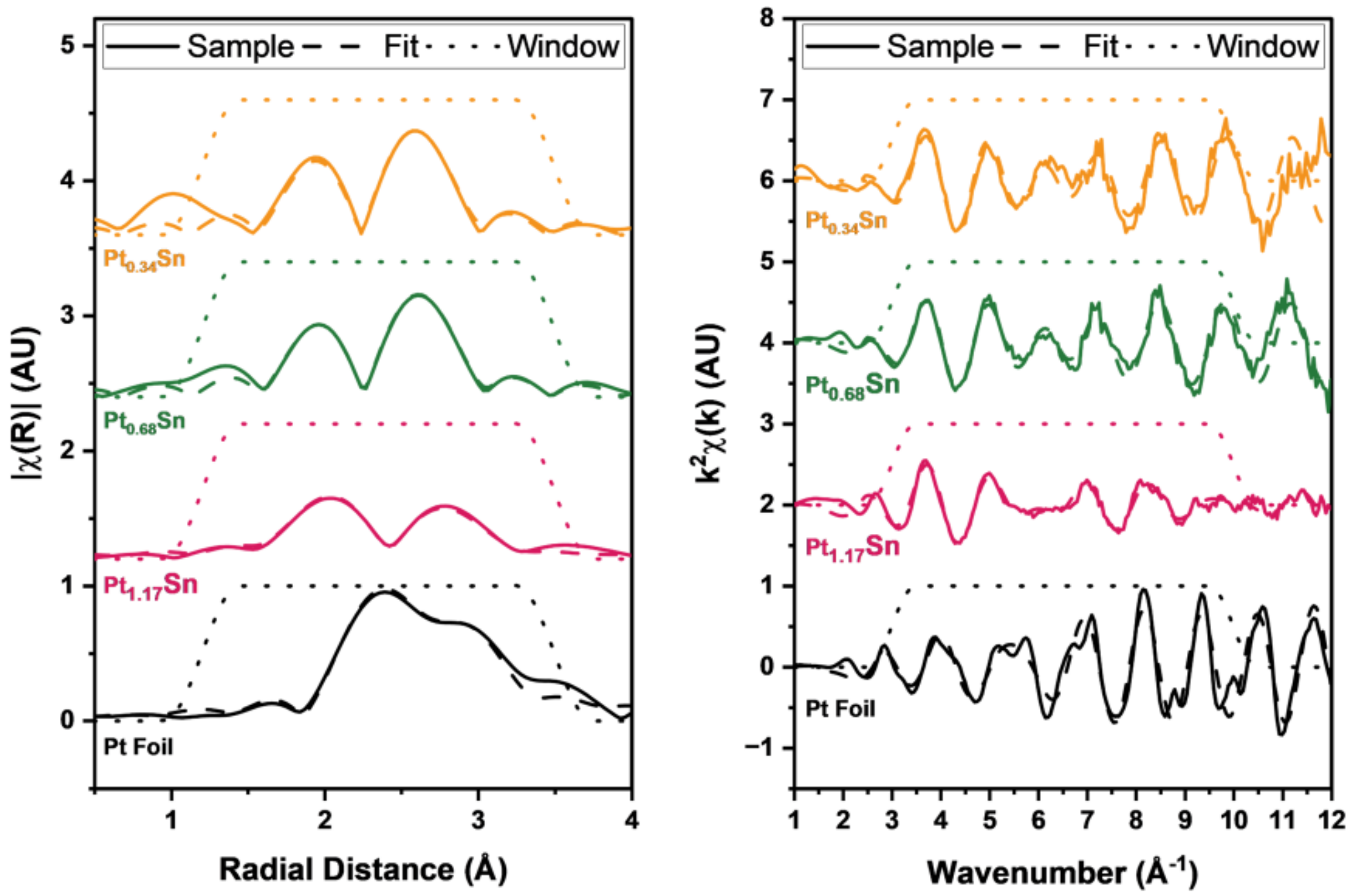

Figure S31. Fitting of Pt-L_3 edge EXAFS spectra with a Pt-Pt and Pt-Sn path.

Table S4. Sn-K and Pt-L_3 XANES edge energies (E_0) and Pt-L_3 EXAFS fitting parameters with the scattering path length (r), mean square relative displacement (Debye-Waller-factor) (σ^2), and change in internal energy alignment (ΔE_0)

Material	Path	Coordination Numbers	*r* (Å)	σ^2 (Å)	ΔE_0 (eV)
$Pt_{1.17}Sn$	Pt-Pt	5.7 ± 0.6	2.78 ± 0.01	0.0075 ± 0.0016	2.34 ± 0.68
	Pt-Sn	3.2 ± 0.3	2.69 ± 0.01		
$Pt_{0.68}Sn$	Pt-Pt	2.2 ± 0.5	2.77 ± 0.03	0.0051 ± 0.0013	3.28 ± 0.98
	Pt-Sn	4.5 ± 0.6	2.69 ± 0.01		
$Pt_{0.34}Sn$	Pt-Pt	2.3 ± 0.9	2.78 ± 0.04	0.0052 ± 0.0022	2.61 ± 1.58
	Pt-Sn	4.7 ± 0.9	2.68 ± 0.02		

5.5 Attempt at Rietveld refinement of pXRD data

An attempt was made to set up a Rietveld refinement or the pXRD data using GSAS-II[17] for $Pt_{1.17}Sn$ and $Pt_{0.68}Sn$ (Figure S32). The crystallite size was set to 4 nm, so no structural parameters were refined. Refined parameters were only: the scale factor, the ratio between the two phases in the $Pt_{1.17}Sn$ pattern and the "background" (24 parameters). Not even lattice constants or zero-point were refined, because the broad, weak reflections and the high background lead to correlation. As a result, no reliable structural parameters or other quantitative information could be obtained from a Rietveld refinement and we decided to use the powder diffraction merely as a tool for semi-quantitative phase identification, based on a comparison of the calculated reflection positions and intensities with

the measured ones. At most we were able to estimate that $Pt_{1.17}Sn$ is composed of 0.49% Pt_3Sn and 0.51% Pt_1Sn_1.

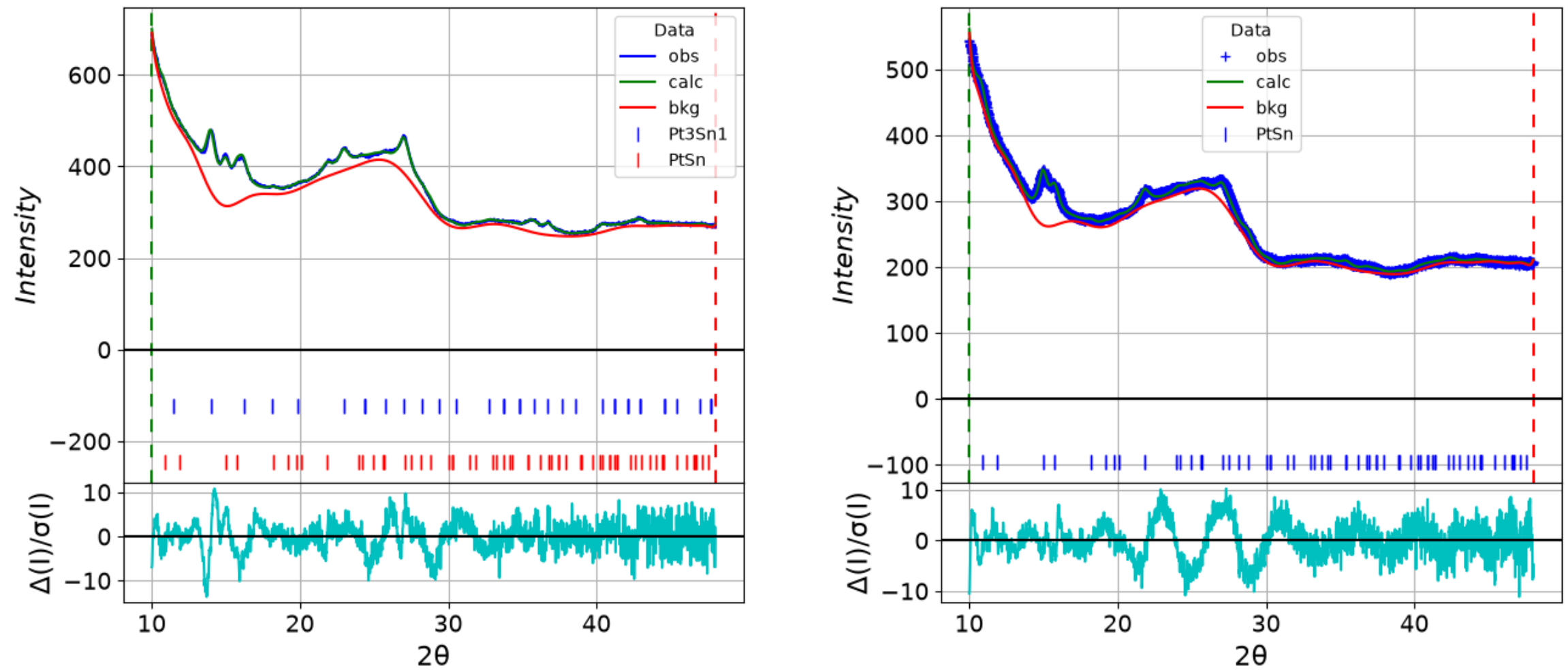

Figure S32. Attempted fitting of background to the pXRD data for $Pt_{1.17}Sn$ (right) and $Pt_{0.68}Sn$ (left) showing that the pattern is primarily dominated by the silica background signal.

6 References

(1) Gsell, R.; Zeldin, M. Synthesis and Spectroscopic Properties of Tin(II) Alkoxides. *J. Inorg. Nucl. Chem.* **1975**, *37* (5), 1133–1137. https://doi.org/10.1016/0022-1902(75)80458-1.

(2) Ehinger, C.; Zhou, X.; Candrian, M.; Docherty, S. R.; Pollitt, S.; Copéret, C. Group 10 Metal Allyl Amidinates: A Family of Readily Accessible and Stable Molecular Precursors to Generate Supported Nanoparticles. *JACS Au* **2023**, *3* (8), 2314–2322. https://doi.org/10.1021/jacsau.3c00334.

(3) Schneider, C. A.; Rasband, W. S.; Eliceiri, K. W. NIH Image to ImageJ: 25 Years of Image Analysis. *Nat. Methods* **2012**, *9* (7), 671–675. https://doi.org/10.1038/nmeth.2089.

(4) Generated Using CrystalDiffract®: A Powder Diffraction Program for Mac and Windows. CrystalMaker Software Ltd, Oxford, England (Www.Crystalmaker.Com).

(5) Zagorac, D.; Müller, H.; Ruehl, S.; Zagorac, J.; Rehme, S. Recent Developments in the Inorganic Crystal Structure Database: Theoretical Crystal Structure Data and Related Features. *J. Appl. Crystallogr.* **2019**, *52* (5), 918–925. https://doi.org/10.1107/S160057671900997X.

(6) Ravel, B.; Newville, M. *ATHENA* , *ARTEMIS* , *HEPHAESTUS* : Data Analysis for X-Ray Absorption Spectroscopy Using *IFEFFIT*. *J. Synchrotron Radiat.* **2005**, *12* (4), 537–541. https://doi.org/10.1107/S0909049505012719.

(7) Dyadkin, V.; Pattison, P.; Dmitriev, V.; Chernyshov, D. A New Multipurpose Diffractometer PILATUS@SNBL. *J. Synchrotron Radiat.* **2016**, *23* (3), 825–829. https://doi.org/10.1107/S1600577516002411.

(8) Origin(Pro) 2024.

(9) Schurko, R. W. Ultra-Wideline Solid-State NMR Spectroscopy. *Acc. Chem. Res.* **2013**, *46* (9), 1985–1995. https://doi.org/10.1021/ar400045t.

(10) Kupce, E.; Freeman, R. Adiabatic Pulses for Wideband Inversion and Broadband Decoupling. *J. Magn. Reson. A* **1995**, *115* (2), 273–276. https://doi.org/10.1006/jmra.1995.1179.

(11) Bhattacharyya, R.; Frydman, L. Quadrupolar Nuclear Magnetic Resonance Spectroscopy in Solids Using Frequency-Swept Echoing Pulses. *J. Chem. Phys.* **2007**, *127* (19), 194503. https://doi.org/10.1063/1.2793783.

(12) Carr, H. Y.; Purcell, E. M. Effects of Diffusion on Free Precession in Nuclear Magnetic Resonance Experiments. *Phys. Rev.* **1954**, *94* (3), 630–638. https://doi.org/10.1103/PhysRev.94.630.

(13) Meiboom, S.; Gill, D. Modified Spin-Echo Method for Measuring Nuclear Relaxation Times. *Rev. Sci. Instrum.* **1958**, *29* (8), 688–691. https://doi.org/10.1063/1.1716296.

(14) Jones, K.; Lappert, M. F. 352. Amino-Derivatives of Metals and Metalloids. Part I. Preparation of Aminostannanes, Stannylamines, and Stannazanes. *J. Chem. Soc. Resumed* **1965**, 1944. https://doi.org/10.1039/jr9650001944.

(15) Sakamoto, K.; Plodinec, M.; Müller, C. R.; Copéret, C. Catalysts Deactivation Models in Propane Dehydrogenation Reaction. June 26, 2026. https://doi.org/10.26434/chemrxiv.15005215/v1.

(16) Virtanen, P.; Gommers, R.; Oliphant, T. E.; Haberland, M.; Reddy, T.; Cournapeau, D.; Burovski, E.; Peterson, P.; Weckesser, W.; Bright, J.; Van Der Walt, S. J.; Brett, M.; Wilson, J.; Millman, K. J.; Mayorov, N.; Nelson, A. R. J.; Jones, E.; Kern, R.; Larson, E.; Carey, C. J.; Polat, İ.; Feng, Y.; Moore, E. W.; VanderPlas, J.; Laxalde, D.; Perktold, J.; Cimrman, R.; Henriksen, I.; Quintero, E. A.; Harris, C. R.; Archibald, A. M.; Ribeiro, A. H.; Pedregosa, F.; Van Mulbregt, P.; SciPy 1.0 Contributors; Vijaykumar, A.; Bardelli, A. P.; Rothberg, A.; Hilboll, A.; Kloeckner, A.; Scopatz, A.; Lee, A.; Rokem, A.; Woods, C. N.; Fulton, C.; Masson, C.; Häggström, C.; Fitzgerald, C.; Nicholson, D. A.; Hagen, D. R.; Pasechnik, D. V.; Olivetti, E.; Martin, E.; Wieser, E.; Silva, F.; Lenders, F.; Wilhelm, F.; Young, G.; Price, G. A.; Ingold, G.-L.; Allen, G. E.; Lee, G. R.; Audren, H.; Probst, I.; Dietrich, J. P.; Silterra, J.; Webber, J. T.; Slavič, J.; Nothman, J.; Buchner, J.; Kulick, J.; Schönberger, J. L.; De Miranda Cardoso, J. V.; Reimer, J.; Harrington, J.; Rodríguez, J. L. C.; Nunez-Iglesias, J.; Kuczynski, J.; Tritz, K.; Thoma, M.; Newville, M.; Kümmerer, M.; Bolingbroke, M.; Tartre, M.; Pak, M.; Smith, N. J.; Nowaczyk, N.; Shebanov, N.; Pavlyk, O.; Brodtkorb, P. A.; Lee, P.; McGibbon, R. T.; Feldbauer, R.; Lewis, S.; Tygier, S.; Sievert, S.; Vigna, S.; Peterson, S.; More, S.; Pudlik, T.; Oshima, T.; Pingel, T. J.; Robitaille, T. P.; Spura, T.; Jones, T. R.; Cera, T.; Leslie, T.; Zito, T.; Krauss, T.; Upadhyay, U.; Halchenko, Y. O.; Vázquez-Baeza, Y. SciPy 1.0: Fundamental Algorithms for Scientific Computing in Python. *Nat. Methods* **2020**, *17* (3), 261–272. https://doi.org/10.1038/s41592-019-0686-2.

(17) Toby, B. H.; Von Dreele, R. B. *GSAS-II* : The Genesis of a Modern Open-Source All Purpose Crystallography Software Package. *J. Appl. Crystallogr.* **2013**, *46* (2), 544–549. https://doi.org/10.1107/S0021889813003531.
